# T cell-redirecting therapies in hematological malignancies: Current developments and novel strategies for improved targeting

**DOI:** 10.1016/j.ymthe.2024.07.028

**Published:** 2024-08-05

**Authors:** Georgina S.F. Anderson, Michael A. Chapman

**Affiliations:** 1 MRC Toxicology Unit, University of Cambridge, Cambridge CB2 1QR, UK; 2 Department of Haematology, University of Cambridge, Cambridge CB2 0XY, UK; 3 Addenbrooke’s Hospital, Cambridge Universities Foundation Trust, Cambridge CB2 0QQ, UK

**Keywords:** CAR T cell, T cell engager, T cell-redirecting therapies, immunotherapy, hematological malignancies, on-target, off-tumor toxicities

## Abstract

T cell-redirecting therapies (TCRTs), such as chimeric antigen receptor (CAR) or T cell receptor (TCR) T cells and T cell engagers, have emerged as a highly effective treatment modality, particularly in the B and plasma cell-malignancy setting. However, many patients fail to achieve deep and durable responses; while the lack of truly unique tumor antigens, and concurrent on-target/off-tumor toxicities, have hindered the development of TCRTs for many other cancers. In this review, we discuss the recent developments in TCRT targets for hematological malignancies, as well as novel targeting strategies that aim to address these, and other, challenges.

## Graphical abstract

**Figure:**
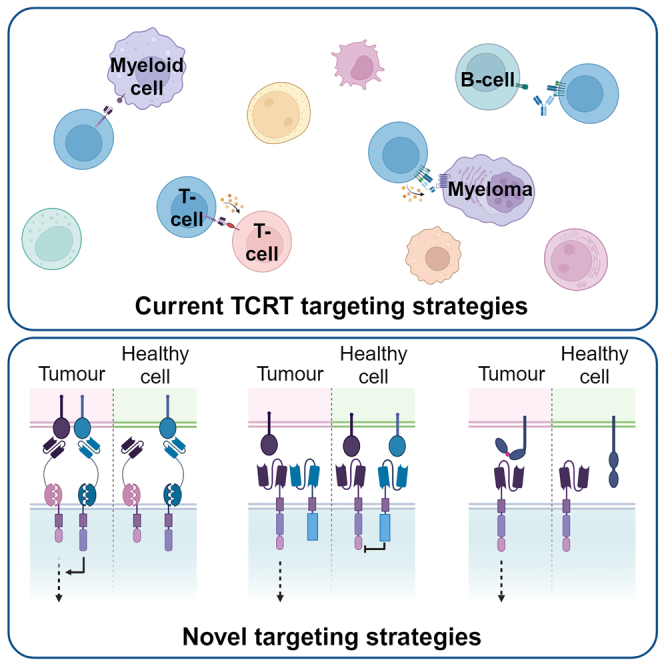

## Teaser

In this review, Chapman and Anderson summarize preclinical and early-stage clinical targets for T cell-redirecting therapies (TCRTs) in hematological malignancies and discuss novel targeting strategies that will improve the specificity of future TCRT therapies to enable safer and more effective treatment.

## Introduction

**Figure 1:**
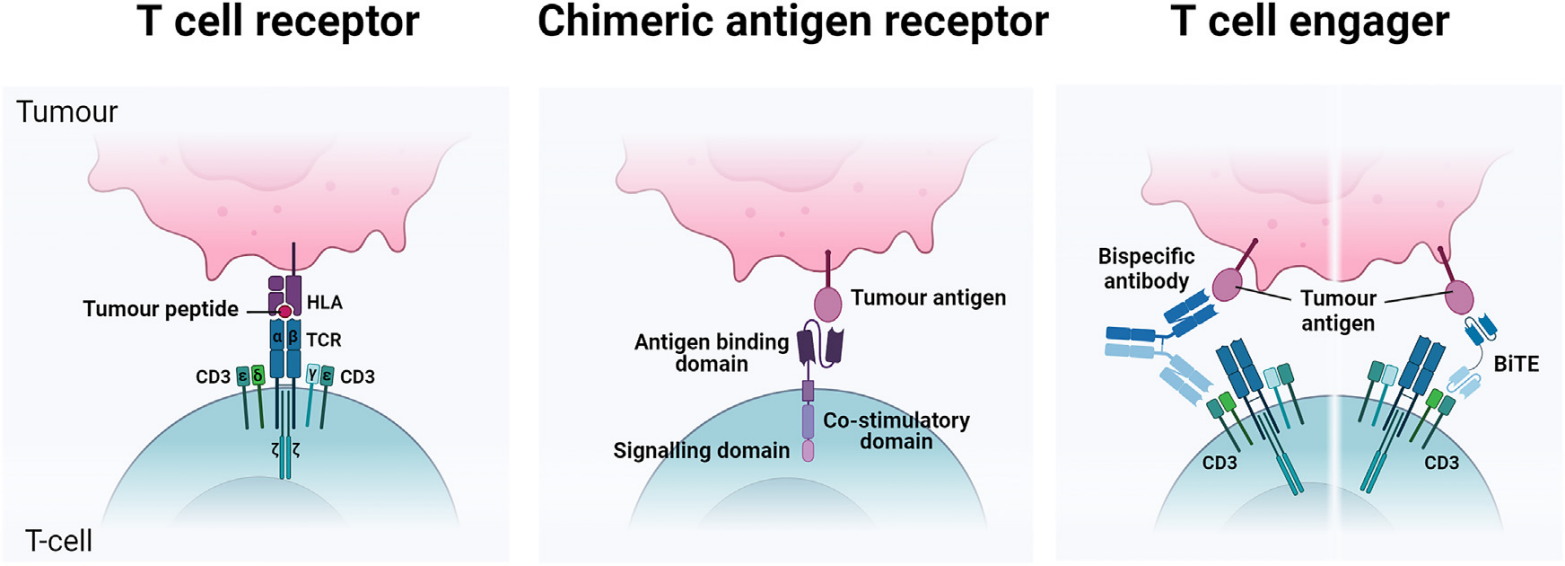
Schematic view of T cell-redirecting therapies The T cell receptor (TCR) complex, composed of two TCR chains (α/β or γ/δ) and six CD3 chains, recognizes peptides presented by the MHC on the target cell. A chimeric antigen receptor (CAR) contains an antigen-binding domain (typically a single-chain variable fragment [scFv]) fused to a co-stimulatory domain (such as CD28 or 4-1BB) and a CD3ζ signaling domain. TCEs: bispecific antibodies or bispecific TCEs (BiTEs) target a tumor antigen and CD3 simultaneously to activate and redirect T cells to tumor cells.

The introduction of immunotherapies has led to a paradigm shift in cancer treatment over the last two decades. The term immunotherapy encompasses a wide range of treatment modalities that use the immune system to help fight cancer. The initial focus was on antibodies. These can block or stimulate cell signaling pathways to induce cell death or reduce proliferation, recruit immune cells to induce cell death, reverse immunosuppression (checkpoint inhibition), or deliver toxins to malignant cells with antibody-drug conjugates (ADCs). T cell-redirecting therapies (TCRTs) have more recently moved into the spotlight. TCRT describes the use of T cell engagers (TCEs), bi- or tri-specific antibodies that can bring T cells and targets into close contact to initiate cell killing, and adoptive cell therapy using chimeric antigen receptor (CAR) or T cell receptor (TCR) T cells that have been genetically engineered to target a specific tumor antigen (Figure 1). CD19- and B cell maturation antigen (BCMA)-targeted CAR-T therapies have proved particularly successful in the treatment of B and plasma cell malignancies, respectively. Loss of lineage-restricted markers, such as CD19 for B cells, is reasonably well tolerated. However, other cancers have proved more challenging to target. In this review, we discuss emerging preclinical and early-phase targets in the TCRT field for hematological malignancies and novel targeting strategies to improve the specificity and efficacy of these therapies.

## TCRTs in hematological malignancies

### What makes an ideal TCRT target?

For a safe and effective TCRT, target selection is paramount. An ideal TCRT target should be consistently expressed across all tumor cells within a patient, including cancer stem cells, to achieve tumor clearance and prevent tumor recurrence. For CAR T cells and TCEs, targets must be localized at the cell surface and should be highly expressed to achieve full T cell activation.^1^ While not essential, expression of TCRT targets should not show significant inter-patient heterogeneity, to maximize the number of patients that can benefit from a given therapy. Off-tumor expression can be tolerated to some degree in non-vital cells, but absent expression in vital tissue is imperative to prevent severe on-target/off-tumor toxicities. To avoid fratricide, which can impair CAR-T cell manufacturing and efficacy,^2^ target antigens should not be expressed on T cells, and although not essential, an ideal target antigen would play a pro-tumorigenic role, such that loss of the antigen is unlikely, or would increase susceptibility to other therapies.

### B cell malignancies

B cell malignancies encompass a large and highly heterogeneous group of cancers that can arise at various stages of the B cell differentiation pathway. Some B cell tumor targets are pan-B cell markers, such as CD19, CD20, and CD22, and the vast majority of TCRT clinical trials target one of these three antigens (clinicaltrials.gov). CD19-targeted therapies have shown unprecedented efficacy, achieving up to 90% complete response (CR) rates in trials for B cell acute lymphoblastic leukemia (B-ALL)^3^^,^^4^ and B cell lymphomas^5^^,^^6^^,^^7^ and there are now four US Food and Drug Administration (FDA)-approved CAR-T cell products targeting CD19. Despite these impressive responses, not all patients will respond, and many fail to achieve long-term remission.^8^ CD19-negative relapses can be treated with CD22 or CD20 TCRTs,^9^^,^^10^ but these antigens are also subject to antigen escape.^11^^,^^12^^,^^13^ Combination therapies against CD19, CD20, and CD22 may improve outcomes,^14^^,^^15^^,^^16^ but it is likely that additional targets will be needed. Possible preclinical and early-phase targets are summarized in Table 1.

**Table 1:** Preclinical and early-phase TCRT targets for B cell malignancies

Target^a^	Reported on-tumor expression	Reported off-tumor expression	Advantages	Disadvantages	Additional notes
BAFF-R	high expression in mature B cell neoplasms (BL, MCL, FL, DLBCL, MZL, B-CLL) and aberrant expression in B-ALL^18^^,^^19^^,^^20^	healthy mature B cells^17^ and low expression in hepatocytes^280^^,^^281^	BAFF-R CAR T cells and mAbs are effective *in vitro* and *in vivo* against patient samples following CD19- and CD20-targeted immunotherapies^20^^,^^21^ pro-survival role in some healthy B cells, which may prevent antigen escape/loss^17^ not on normal pre-B cells, which may reduce the severity of B cell aplasia compared to CD19-targeted TCRTs^18^	low or varied expression in immature B cells could restrict application in some B cell neoplasms^17^ off-tumor expression poses a risk for hepatotoxicity and will cause B cell aplasia^17^^,^^280^^,^^281^	CAR T cells using BAFF as the antigen recognition domain can target all three BAFF receptors (BAFF-R, TACI and BCMA), which may mitigate the risk of antigen escape and broaden patient applicability, but would also cause plasma cell depletion^17^^,^^281^ BAFF-R targeting mAbs have been well tolerated, demonstrating potential safety for this target^282^ phase I trials for TCRTs are ongoing. Preliminary results from a phase I CAR T cell are encouraging with a 100% ORR in the three patients reported thus far^22^
CD79ab	MALT, DLBCL, MCL, FL, BL, and MZL^25^^,^^26^^,^^27^ low expression in CLL^25^	healthy B cells^25^ and immature myeloid cells (CD79a)^283^	highly restricted to the B cell lineage, limiting OTOT toxicities^23^ CD79a and b expression is retained in patient samples after CD19- and CD22-targeting TCRTs^25^^,^^27^^,^^284^ pro-survival role in some lymphomas may prevent antigen escape/loss^23^	off-tumor expression will cause B cell aplasia^23^ target of interest for mature B cell neoplasms only^25^^,^^26^^,^^27^	the FDA approval of a CD79b-ADC (polatuzumab vedotin) for the treatment of DLBCL, supports the safety and efficacy of CD79-TCRTs^28^ phase I/II trials for TCRTs ongoing with results pending
CD37	DLBCL, BL, MCL, FL, and MZL^30^^,^^285^^,^^286^^,^^287^	healthy B cells and minimal monocyte expression^30^^,^^288^	high and homogeneous expression across B-NHL subtypes^286^^,^^288^	target of interest for mature B cell lymphomas only^287^ off-tumor expression will result in B cell aplasia^288^ a case of CD37 antigen loss following CD37-CAR-T cell therapy has already been reported^36^	early-phase clinical trials of antibody-based therapies targeting CD37 had limited efficacy^31^^,^^32^^,^^33^ clinical trials are ongoing and suggest potential efficacy for CD37-CAR T cells (2 CR, 1 PR, and 1 PD) but two cases of prolonged pancytopenia with marrow aplasia is of concern^36^ CD37 is also aberrantly expressed in some T cell malignancies^30^
CD72	B-ALL and B-NHL^289^ particularly high expression in MLLr B-ALL^38^	healthy B cells^38^	higher expression in DLBCL than CD22 which may improve efficacy^9^^,^^38^ expressed on all subtypes of B-ALL ^38^ CD72 loss may increase sensitivity to chemotherapy through decreased adhesion within the bone marrow niche^38^ low risk for OTOT toxicities^38^ CD72-CAR T cells were effective in a preclinical model of CD19^low/neg^ relapse^38^	CD72^low^ relapse has been reported in preclinical xenograft models^290^	may also be a target of interest for AML^289^ not in clinical trials yet but Temple et al. suggest that their CD72-CAR T cell will be progressed to the clinic^290^ SHIP-1 inhibitors can increase CD72 expression, providing a rational combination strategy to improve efficacy^38^
ROR1	DLBCL, CLL, MCL, and a subset of B-ALL^40^^,^^291^^,^^292^	absent in all B cells except a subset of normal B cell precursors^40^ adipose tissue, pancreas, gastrointestinal tract, parathyroid glands, and lung^40^^,^^293^^,^^294^	mature B cells would be spared, providing some short-term protection of humoral immunity^40^ side-population CLL cells, a chemo-resistant population, are sensitive to ROR-1 CAR T cells^40^ Increased expression in CD19-TCRT relapsed MCL patients^292^	risk for the long-term depletion of immature B cells^40^ non-lymphoid tissue expression poses a serious OTOT toxicity risk and lethal OTOT toxicity has been reported in preclinical ROR1-CAR-T cell xenograft models^42^^,^^294^ restricted to more mature B cell neoplasms^40^^,^^291^^,^^292^	clinical experience with the ROR1-ADC (zilovertamab vedotin [VLS-101]) in CLL and B-NHL, with no unexpected toxicities reported in a phase I trial^295^ preliminary results from ROR1 CAR T cells for solid malignancies and a ROR1 bispecific TCE in R/R MCL/CLL also suggest safety.^296^^,^^297^^,^^298^ However, a grade 5 AE (consistent with CRS and ICANS) in a separate ROR1-CAR-T cell trial warrants caution.^299^ Efficacy is promising thus far^298^^,^^300^
TSPLR	TSLPR-overexpressing Philadelphia-like B-ALL^43^^,^^44^	dendritic cells, subset of T cells and monocytes. Cytoplasmic staining in the kidney, colon, liver, and skin^43^^,^^44^	highly expressed in a high-risk prognosis that has a high-rate of relapse and poor-response to chemotherapy^301^ pro-tumorigenic role in B-ALL may prevent antigen escape/loss^302^	the low expression on other immune cell subsets may pose an OTOT toxicity risk^43^ restricted expression to a small subset of B-ALL patients^43^	TSLPR-CAR T cells demonstrated comparable efficacy to CD19- and CD22-CAR T cells in *in vivo* xenograft models^43^
Light chain (kappa or lambda)	late-stage immature/mature B cell neoplasms (B-NHL, CLL/SLL and MM)^46^^,^^303^	late-stage immature/mature B cells expressing the target light chain (approximately half)^46^^,^^303^	expressed in the majority of B-NHL subtypes^46^ a substantial proportion of healthy B cells would be spared, preserving humoral immunity and reducing the risk of severe infections compared to pan-B cell CAR T cells^303^ free immunoglobulins may provide low tonic-signaling that may promote CAR-T cell persistence^303^ pro-survival role for BCR signaling in some lymphomas may prevent antigen escape/loss^23^	possible loss of immune responses against particular epitopes (although this should be compensated for by reciprocal light chain Ig against different epitopes)^303^ expression restricted to mature B cell neoplasms^46^^,^^303^	kappa-light-chain phase I clinical trial safety results are encouraging, albeit with modest efficacy (2 CR, 1 PR, 1 SD, and 5 NR). This may be partly due to the lymphodepletion regime prior to infusion^45^ lambda-light-chain CAR T cells may be particularly beneficial for MCL, which is more commonly lambda-light-chain positive than kappa^46^
IGHV4-34	subset of late-stage immature/mature B cell neoplasms (B-NHL, CLL)^47^^,^^304^	late-stage immature/mature B cells expressing IGHV4-34 (∼5%)^47^	the majority (∼95%) of healthy B cells should be spared^47^	although IGHV4-34 is commonly expressed in some subtypes (DLBCL, vitreoretinal lymphomas, HCL, and CLL), IGHV4-34 TCRTs would be highly restricted to a small subset of B cell neoplasms^47^^,^^304^	IGHV-34 is also a target of interest for systemic lupus erythematosus^305^ still at the preclinical stage
CD70	DLBCL, FL, HL, MM, and WM^306^	subset of activated B and T cells and dendritic cells^306^	CD70 CAR T cells have shown preclinical activity *in vivo* against CD19^neg^ target cells^307^ low risk for OTOT toxicities: transient off-tumor expression that is restricted to a subset of activated immune cells^306^	elimination of CD70-positive T cells could impair T cell-mediated immunity, including anti-EBV responses^308^	CD70-targeting ADCs have shown limited efficacy, and their clinical application is limited by frequency and severity of thrombocytopenia.^309^ However, this is likely due to treatment modality.^309^ B and T cell aplasias were not reported^309^ clinical trials are ongoing with results pending also a target of interest for AML, T-ALL, and MM^306^^,^^310^
CD74	B-NHL, HL, CLL, MM, and WM^134^^,^^311^^,^^312^^,^^313^	healthy B cells, monocytes, dendritic cells, subset of myeloid cells, and subset of T cells^134^^,^^312^	CD74-CAR T cells were effective *in vitro* against a post-CD19-TCRT relapse patient sample^313^ pro-survival role in B cells which may prevent antigen escape/loss^313^	expression on healthy B cells as well as other immune cells may be a risk for cytopenias^313^^,^^314^	CD74-targeting mAbs and ADCs have shown safety but limited efficacy in clinical trials^315^^,^^316^^,^^317^
CD32b	CLL/SLL, MCL, and SMZL^318^^,^^319^	healthy B cells, subset of T- and dendritic- cells^319^^,^^320^ non-lymphoid tissue: airway smooth muscle cells, liver sinusoidal endothelial cells, Kupffer cells and placenta^319^	CD32b mediates resistance to rituximab by antibody internalization. Therefore, loss of target antigen could increase sensitivity to rituximab^321^ higher and more uniform than CD19 in CLL^319^	risk for OTOT toxicities toward non-lymphoid tissue and B cells^319^ T cell expression may lead to some CAR-T cell fratricide^320^ only approximately half of B-NHL are positive for CD32b, which would limit therapeutic applicability^318^	–

a AE, adverse event; ALL, acute lymphoblastic leukemia; BCR, B cell receptor; BL, Burkitt lymphoma; cHL, classical Hodgkin lymphoma; CLL/SLL, chronic lymphocytic leukemia/small lymphocytic lymphoma; CR, complete response; CRS, cytokine release syndrome; DLBCL, diffuse large B cell lymphoma; FL, follicular lymphoma; HL, Hodgkin lymphoma; HSPC, hematopoietic stem and progenitor cell; ICANS, Immune effector cell-associated neurotoxicity syndrome; mAb, monoclonal antibody; MALT, mucosa-assisted lymphoid tissue lymphoma; MCL, mantle cell lymphoma; MM, multiple myeloma; MZL, marginal zone lymphoma; NHL, non-Hodgkin lymphoma; NR, no response; ORR, overall response rate; OTOT, on target, off tumor; PD, progressive disease; PR, partial response; R/R, relapsed/refractory; SD, stable disease; SMZL, splenic marginal zone lymphoma; TCE, T cell engager; TCRT, T cell-redirecting therapy; WM, Waldenstrom macroglobulinemia.

One of the most promising recent targets is the B cell-activating factor receptor (BAFF-R), which plays a key role in B cell viability, development, and survival.^17^ BAFF-R is also highly expressed in mature B cell neoplasms^18^^,^^19^ and expression is retained after relapse with CD19- and CD20-targeted therapies,^20^^,^^21^ making BAFF-R an attractive target in B cell disease. Even though BAFF-R is expressed at lower levels than CD19, anti-BAFF-R CAR T cells have shown preclinical efficacy against a wide range of lymphoma and chronic lymphocytic leukemia (CLL) cell lines.^20^ They are now in early-phase clinical trials, with promising initial results.^22^

The B cell receptor is a protein complex formed of surface immunoglobulin and its signaling component CD79, a heterodimer of CD79a and CD79b.^23^ These two proteins are restricted to the B cell lineage, are highly expressed in the majority of B cell lymphomas, and play a pro-survival role that can drive tumorigenesis.^24^^,^^25^^,^^26^^,^^27^ CD79b is the most clinically advanced target of the two and an anti-CD79b ADC (polatuzumab vedotin, CD79b-MMAE) has been FDA approved for the treatment of diffuse large B cell lymphoma (DLBCL), validating CD79 as a safe and effective target.^28^ Several CD79-targeting TCRT trials are currently ongoing with results pending (clinicaltrials.gov).

CD37 is another target of interest for mature B cell neoplasms.^29^^,^^30^ Although early-phase clinical trials of antibody-based therapies have proved disappointing, with several terminated early by their sponsor,^31^^,^^32^^,^^33^^,^^34^^,^^35^ it is possible that CAR T cells may fare better, with impressive preclinical^30^ and preliminary clinical data.^36^ One potential concern with targeting CD37 is off-tumor expression on monocytes,^37^ raising the prospect of on-target/off-tumor toxicity.

Excluding CD19 and CD22, CD72 is the only target known to be expressed across all B-ALL subtypes. Although currently only at the preclinical stage, Investigational New Drug approval for a nanobody-based CAR targeting CD72 is underway.^38^ CD72 is particularly highly expressed in the poor-prognosis MLLr B-ALL subtype, which is less responsive to more classic CAR-T cell targeting.^38^

Although these targets will expand the therapeutic repertoire in the post-CD19-relapse landscape, a particular challenge remains unaddressed (i.e., frequent B cell aplasia). That loss of healthy B cells can be clinically managed using immunoglobulin (Ig) infusions has facilitated the rapid advancements of TCRTs in this disease area. Although it is considered clinically tolerable, B cell aplasia can persist for several years post CAR-T cell infusion^39^—beyond the usual nonspecific cytopenias associated with CAR-T cell therapy—putting patients at an increased risk for severe infections. Identifying target antigens with minimal expression on healthy B cells would be highly desirable.

The oncofetal protein receptor tyrosine kinase-like orphan receptor 1 (ROR1) may enable more selective targeting. ROR1 is differentially expressed between normal and malignant B cells, and preclinical studies suggest that ROR1-CAR T cells selectively kill CLL cells while sparing resting and activated B cells.^40^ While this offers the potential of fewer infections compared to pan-B cell targets, the potential long-term loss of immature B cells and low-level expression in non-hematological cells may present a risk of other on-target/off-tumor toxicities.^40^^,^^41^^,^^42^

TSLPR (*CRLF2*) overexpression due to gene rearrangements is a frequent occurrence in the poor-prognosis Philadelphia chromosome-like (Ph-like) ALL subtype.^43^ *In vivo* studies have shown efficacy of both bispecific TCEs and CAR T cells,^43^^,^^44^ but results from an ongoing trial will be imperative to establish on-target/off-tumor toxicities. Further, TSLPR is highly restricted to a small subset of B-ALL, which will limit widespread utility of this target.^43^

Finally, the clonal nature of some B cell malignancies may offer a strategy to minimize B cell aplasia. Targeting the light chain (kappa or lambda) provides high specificity for the clonal malignant cells while sparing a proportion of the healthy B cells expressing the reciprocal light chain.^45^ Kappa-CAR T cells have shown safety and feasibility in mature B cell malignancies. Efficacy was limited but this may be in part due to the absence of a strong lymphodepleting regime.^45^ Preclinical evidence suggests lambda-light-chain CAR T cells would be similarly well tolerated.^46^ Similarly, the IGHV4-34 heavy-chain-variable gene is frequently expressed in a proportion of clonal mature B cell malignancies but only in ∼5% of the normal B cell repertoire.^47^ Other TCRT targets of interest but not discussed further herein include CD70, CD74, and CD32b (Table 1).

### Multiple myeloma

Multiple myeloma (MM), a plasma cell malignancy, has seen dramatic improvements in survival over the last two decades with the advent of immunomodulatory agents, proteasome inhibitors, and anti-CD38 targeted monoclonal antibodies.^48^ Despite these advancements, responses are not typically durable, and patients will eventually relapse and become refractory to treatment. Like B cell malignancies, myeloma is well suited to immunotherapy as healthy plasma cell loss is reasonably well tolerated. BCMA (TNFRSF17) is the target that has paved the way in myeloma, achieving an impressive 81% overall response rate (ORR) in a first-in-human trial.^49^ Follow-up analyses have confirmed similar results across multiple trials^50^ and there are now four FDA-approved BCMA-targeted immunotherapies: two CAR T cells and two bispecific TCEs. Nonetheless, most patients still progress after BCMA-targeted therapy^49^^,^^51^^,^^52^^,^^53^ and additional targets for TCRTs will be required to maintain durable remissions, or even cure. Potential targets are summarized in Table 2.

**Table 2:** Preclinical and early-phase TCRT targets for myeloma

Target^a^	Reported on-tumor expression	Reported off-tumor expression	Advantages	Disadvantages	Additional notes
GPRC5D	MGUS, SMM, PCL, and MM^59^	healthy PCs.^59^ hair follicles, nail beds, filiform papillae of the tongue and inferior olivary nucleus^62^^,^^63^	highly expressed in myeloma with no B cell expression and minimal PC expression. This may reduce the severity and/or frequency of infections as seen with BCMA-TCRTs.^58^^,^^322^ Clinical trial results support this, with lower rates of severe infections^54^^,^^55^^,^^56^^,^^57^^,^^62^ GPRC5D TCRTs have shown efficacy in patients with prior BCMA-targeting therapies exposure^55^^,^^56^^,^^62^ as a GPCR, exposed epitopes are likely to be membrane-proximal, which may enable the formation of more efficient immune synapses to enhance anti-tumor efficacy^59^	high frequency of nail- and skin-related AEs and dysgeusia in clinical trials due to off-target expression.^55^^,^^56^ OTOT expression may also be the reason for the cerebellar toxicities reported for two GPRC5D-CAR-T cell products^54^^,^^62^ GPRC5D^low/neg^ progressive disease has reported in patients receiving CAR T cells and TCEs^52^^,^^62^	clinical trial results have shown promising efficacy, with both TCEs and CAR T cells achieving >70% ORRs across multiple trials^54^^,^^55^^,^^56^^,^^57^^,^^62^^,^^323^ talquetamab (GPRC5DXCD3 bispecific TCE) recently received accelerated approval and conditional marketing authorization in Europe for triple-class exposed R/R MM^60^^,^^61^ although cerebellar toxicities have been reported for two CAR-T cell products, no neural toxicities have been reported for a third CAR-T cell product or any bispecific TCEs^54^^,^^55^^,^^56^^,^^57^^,^^62^
FCRL5/FCRH5	MGUS, MM (and HCL, CLL, and MCL)^64^^,^^67^	B lineage: pre-B cell to PC^64^^,^^67^	low risk for OTOT with no know expression outside the B cell compartment^67^ lower expression on B cells and healthy PCs may limit cytotoxicity toward these cells^64^^,^^66^^,^^67^ higher and more uniform expression than BCMA, and expression is retained in patients post BCMA TCRT.^66^^,^^67^ FCRL5XCD3 TCEs have shown efficacy in patients who have previously received BCMA-targeted immunotherapies^69^ expression is associated with 1q21 gain, a poor prognostic marker in MM^65^^,^^66^^,^^67^	cleavage of FCRL5 could provide a means for antigen escape and may impair CAR-T cell cytolytic activity^67^	phase I results for an FCRL5-ADC (DFRF4539A, NCT01432353) were disappointing with two (5%) PR, one (3%) MR, and 18 (46%) SD as best response.^68^ TCRTs may be a more effective means to target FCRH5 as suggested by early clinical results^69^^,^^71^ early results suggest lower response rates for FCRL5-TCEs than GPRC5D- and BCMA-TCEs (54.5% ORR at the 160-mg dose level, NCT03275103) but responses are durable^69^^,^^71^ may also be a target of interest for MCL, HCL, and CLL^67^
SLAMF7 (CS1)	MGUS, SMM, MM^324^	pro-B cells, plasma cells, NK cells, T cells, activated monocytes, dendritic cells^324^^,^^325^	SLAMF7 is expressed in all MM patients and is retained in relapsed disease, including post BCMA-CAR-T cell therapy^51^^,^^324^^,^^325^ pro-tumorigenic^326^	SLAMF7 is expressed on nearly all CD8+ T cells, resulting in fratricide^325^ SLAMF7 can be cleaved, providing a mechanism for antigen escape, and CAR-T cell binding of soluble protein may limit efficacy^327^ Off-tumor expression on other immune cell subsets poses a serious risk for lymphopenia^325^	the FDA-approved SLAMF7-targeting mAb (elotuzumab) has shown anti-MM activity when used in combination for R/R MM, but activity in newly-diagnosed MM is limited^328^ SLAMF7-CAR-T cell trials are currently ongoing with results pending. Results from a bispecific BCMA-SLAMF7 CAR-T cell trial suggest that SLAMF7-targeting does not increase the rate of infections compared to BCMA CAR T cells alone, but these bispecific CAR T cells do have reduced cytolytic activity^329^
Kappa-light chain	late-stage immature/mature B cell neoplasms (B-NHL, CLL/SLL) and kappa-restricted MM^46^^,^^303^	late-stage immature/mature B cells expressing the kappa-light chain (approximately half)^303^	CAR T cells selectively eliminate clonal malignant cells while sparing normal B cells with the reciprocal light chain (approximately half)^45^^,^^303^ although surface immunoglobulin is minimal on MM cells, surface immunoglobulin is expressed on myeloma-initiating cells^330^	kappa-light-chain immunoglobulins are secreted by myeloma cells, and so the low surface expression may limit efficacy. In addition, the high level of secreted immunoglobulins in MM might lead to excessive stimulation and exhaustion^45^	in a phase I trial for kappa-CAR T cells for NHL/CLL and MM, modest anti-myeloma effects were observed (four of seven achieving SD as best response)^45^ KMA is a membrane-bound form of kappa-light chain found in kappa-restricted MM. KappaMab (MDX-1097), a KMA-targeting mAb, demonstrated efficacy in a phase IIb trial, which may support the targeting of KMA instead of surface kappa Ig for MM^331^
CD229	B-NHL, MM, and PCL^72^^,^^73^^,^^74^^,^^332^	NK cells, mature B and T cells, and plasma cells^72^^,^^74^^,^^332^	expression is highly restricted to the hematopoietic compartment^72^ pro-survival role may reduce the risk for antigen escape^72^ expressed on the chemo-resistant myeloma-initiating/propagating cells^73^^,^^74^	OTOT expression on other immune cells will likely result in cytopenias. CD229 is downregulated following CD3/CD28 stimulation, which may limit CAR-T cell fratricide during manufacturing, but it is currently unknown if this downregulation will be sustained post infusion^74^ soluble CD229 (sCD229) is increased in advanced disease.^332^ It is currently unknown if CAR T cells recognize sCD229 but it may abrogate activity	affinity-tuned CD229 CAR T cells retain anti-myeloma activity but lack cytolytic activity toward healthy lymphocytes^75^
CD1d	MGUS and MM^333^	antigen-presenting cells, B cells, epithelial cells, thymocytes, activated T cells, and HSCs^334^^,^^335^^,^^336^^,^^337^	CD1dXVδ2 bispecific Vγ9Vδ2-TCE can recruit both NKT- and Vγ9Vδ2 T cells, which preferentially target malignant cells over healthy cells (reducing the risk for OTOT toxicities) and have a lower risk for CRS^334^	high risk for antigen escape as CD1d ligation induces B cell and PC death, and expression is lost with disease progression^333^^,^^334^ Expressed on *in vitro* activated T cells which may preclude CAR-T cell development^335^	low expression in advanced disease may limit efficacy in the R/R patient populations likely to constitute early-phase clinical trial cohorts^334^ CD1d is also a target of interest for (myelo)monocytic AML and CLL^334^ early clinical trial results for a CD1dXVδ2 TCE suggest limited efficacy in MM/CLL (disease stabilization in two of eight patients)^338^
SEMA4A	MGUS, SMM, and MM^79^^,^^80^	monocytes, granulocytes, healthy PCs, and a subset of T cells^79^	pro-survival role in MM may reduce the risk for antigen escape^79^ SEMA4A is expressed in the majority (>90% of patients) and is retained in advanced R/R disease. Expression is higher than BCMA, FCRL5, and GPRC5D^79^^,^^339^	OTOT expression may pose a risk for cytopenias. However, expression is much lower than malignant cells and no cytopenias were seen in a murine toxicity model using a cross-reactive SEMA4A-ADC^79^ increased expression in T cells post activation poses a risk for fratricide during manufacturing but does not appear to affect cytolytic capabilities *in vitro*^339^	–
CD46 (MCP)	MGUS, SMM, MM^78^	PC, monocytes, granulocytes, placenta, and prostate^78^	expressed in myeloma-initiating cells^76^ higher expression in MM than healthy PCs may reduce the risk for hypoglobulinaemia^78^ expression is associated with 1q21 gain, a poor prognostic marker in MM^78^	moderate monocyte and granulocyte expression may pose a risk for cytopenias^78^	phase I results of a CD46-ADC (FOR46) have shown modest efficacy (three PR in six patients) with severe cytopenia (3 Gr four neutropenia and one Gr 4 thrombocytopenia)^77^
ILT3 (LILRB4)	MM^80^^,^^82^	monocytes, macrophages, and dendritic cells^80^	an ILT3XCD3 bispecific TCE has shown activity against samples from relapsed patients post BCMA-CAR-T cell therapy^80^ ILT3 is a negative immune receptor and can suppress T cell proliferation in MM and AML, providing a strong rationale for therapeutic targeting^121^	OTOT expression on other immune cells may pose a risk for cytopenias^80^	also a target of interest for monocytic AML^340^
CCR10	MM^82^	healthy PCs, T cells^82^	low risk for OTOT toxicities as minimal expression on other hematopoietic cell subsets^82^ Expression is increased in R/R advanced disease^82^	CCR10 is upregulated on activated T cells, which resulted in fratricide and CAR-T cell manufacturing difficulties in a preclinical study of an anti-CCR10-CAR T cell^82^	–

a AEs, adverse events; GPCR, G-protein-coupled receptor; HCL, hairy cell leukemia; KMA, kappa myeloma antigen; MGUS, monoclonal gammopathy of undetermined significance; MR, minimal response; PC, plasma cell; PCL, plasma cell leukemia; SMM, smoldering multiple myeloma. Other abbreviations as in Table 1.

Recently, GPRC5D has become one of the most prominently targeted surface proteins in myeloma.^54^^,^^55^^,^^56^^,^^57^ Hematopoietic expression is tightly restricted to plasma cells, and, unlike BCMA, expression is much greater in malignant cells compared to their healthy counterparts.^58^ As a G-protein-coupled receptor, it is postulated that GPRC5D is less likely to be shed, cutting off one means of antigen escape, and that the exposed epitopes will be closer to the membrane surface and enable the formation of more efficient immunological synapses between targets and T cells.^59^ Talquetamab, a GPRC5D bispecific TCE, has recently received accelerated FDA approval and conditional marketing authorization in Europe for relapsed/refractory myeloma^60^^,^^61^ on the back of impressive efficacy in a phase I/II trial (>70% ORR).^56^ Forimtamig, a second GPRC5D bispecific TCE, also has promising clinical efficacy,^55^ while GPRC5D-targeting CAR T cells have shown very encouraging results in early-phase trials.^54^^,^^57^^,^^62^ Unfortunately, expression of GPRC5D has been demonstrated outside the immune system, including in hair follicles, nail beds, filiform papillae of the tongue, and potentially the inferior olivary nucleus,^54^^,^^55^^,^^56^^,^^62^^,^^63^ and predictable on-target/off-tumor side effects have been observed in these clinical studies.^54^^,^^55^^,^^56^^,^^62^

FCRL5 (FCRH5) expression is also higher in malignant cells compared to healthy plasma cells and is minimally expressed on B cells.^64^^,^^65^^,^^66^ Expression is not correlated with BCMA and is generally higher and more consistent, making this another potential target in the post-BCMA landscape.^66^^,^^67^ Activity as an ADC target was underwhelming,^68^ but TCRTs often show greater clinical efficacy than ADCs, and FCRL5 has re-emerged as an effective TCE target. Response rates appear lower than GPRC5D- and BCMA-targeted TCEs, but responses were durable.^56^^,^^69^^,^^70^^,^^71^ Investigation into FCRL5 as a CAR-T cell target is currently at the preclinical stage but is showing promise, including in BCMA-negative disease.^66^^,^^67^ While FCRL5 and GPRC5D TCRTs have exhibited impressive anti-myeloma responses, especially in patients with prior BCMA therapy exposure, GPRC5D^low/neg^ progressive disease has already been documented,^52^^,^^62^ and there is the potential for antigen escape through FCRL5 cleavage.^67^

With its anti-apoptotic role in MM and expression on potential MM-initiating/propagating cells, CD229 (LY9) may be a promising candidate target for inducing more durable remissions.^72^^,^^73^^,^^74^ However, expression on other hematopoietic cells may require affinity optimization to mitigate off-tumor toxicities.^75^ CD46 is another target present on myeloma-initiating cells,^76^ and a CD46-ADC has shown modest anti-myeloma activity in a phase I trial,^77^ but, again, moderate monocyte and granulocyte expression may necessitate additional engineering strategies to prevent longer-term cytopenias than are seen with other TCRTs.^77^^,^^78^ Although myeloma cells typically do not express surface immunoglobulin, it has been reported that kappa-restricted myeloma-initiating cells may do, providing a rationale for kappa-CAR T cells in myeloma. Clinically, kappa-CAR T cells have shown limited efficacy, only achieving stable disease in four patients as best response.^45^ This is likely because the low surface expression of the target and kappa-CAR T cells may fare better in combination therapy to enable the eradication of both malignant cells and initiating cells. We, and others, recently identified SEMA4A as a novel myeloma immunotherapeutic target using cell-surface proteomics.^79^^,^^80^ SEMA4A expression is essential for normal myeloma growth *in vitro*, suggesting a reduced risk for antigen escape.^79^ SEMA4A is expressed in other hematopoietic cells, but at a considerably lower level than in myeloma, and we did not see any cytopenias in a murine toxicity model.^79^ Other cell-surface proteomic studies have revealed ILT3 (LILRB4) and CCR10 as additional TCRT targets of interest for myeloma^81^^,^^82^ (Table 2).

### Acute myeloid leukemia

Acute myeloid leukemia (AML), a malignancy of myeloid stem cells, is the most common form of adult acute leukemia. Standard care is chemotherapy, and, although most patients achieve complete remission, this response is often not durable, leading to relapse with chemo-resistant disease.^83^ Allogeneic hematopoietic stem cell (HSC) transplant (allo-HSCT), which exploits graft-versus-tumor cytotoxicity, was an early form of immunotherapy that is still utilized commonly in treatment. However, developing targeted immunotherapies for this disease has been challenging. This is partly a result of disease heterogeneity,^84^ but also because potential antigen targets are also expressed by hematopoietic stem and progenitor cells (HSPCs),^84^^,^^85^ whose long-term loss is less well tolerated than the B cell aplasia seen with CD19-targeted TCRTs. As a result, current TCRTs are predominantly being investigated as a bridge to transplant. Several such novel AML targets are summarized in Table 3.

**Table 3:** Preclinical and early-phase TCRT targets for AML

Target^a^	Reported on-tumor expression	Reported off-tumor expression	Advantages	Disadvantages	Additional notes
CD33	AML bulk cells and LSCs^84^	myeloid progenitor cells, neutrophils, macrophages, T cells, dendritic cells, Kupffer cells, and hepatocytes^84^^,^^86^	expressed in most AML patients, on both bulk cells and LSCs^84^ CD33 expression is retained in relapsed disease^341^	high risk for OTOT toxicities due to expression on hematopoietic cells, including CD34+CD38+ progenitor cells^84^ severe hepatotoxicity and fatal cytopenias have been reported using CD33-ADCs^86^^,^^87^^,^^88^ risk for CAR-T cell fratricide due to low T cell expression^84^	gemtuzumab ozogamicin, a CD33-ADC, received accelerated approval in 2000, but was withdrawn in 2010 due to serious adverse events. Gemtuzumab ozogamicin was approved again in 2017 KO of CD33 (discussed later) in CD34+ HSPCs prior to SCT may provide a means of safely targeting this antigen^267^ limited efficacy for CD33-targeting TCRTs in clinical trials^89^^,^^90^^,^^91^^,^^94^^,^^342^^,^^343^
CD123	AML bulk cells and LSCs^84^	HPCs, monocytes, granulocytes, and endothelial cells^106^^,^^344^	expressed in most AML patients, on both bulk cells and LSCs^84^ pro-survival role in AML^95^ lower expression on HPCs than AML blasts may provide protection^97^^,^^98^^,^^195^	expression on myeloid progenitors, monocytes, granulocytes pose a risk for severe myelotoxicity.^96^ OTOT expression on endothelial cells may cause serious adverse events (capillary leak syndrome and severe CRS)^106^	limited efficacy and severe CRS for CD123-targeting TCEs.^99^^,^^100^^,^^101^ CAR-T cell efficacy may be greater^102^^,^^103^ but severe adverse events (two grade 4 capillary leak syndromes and a grade 5 CRS) pose a serious concern.^104^^,^^105^
CLL-1 (CLEC12A)	AML bulk cells and LSCs^84^^,^^107^	myeloid progenitor cells, monocytes and granulocytes ^107^^,^^108^	expressed in most AML patients, on both bulk cells and LSCs^84^^,^^108^ restricted to the myeloid-lineage, minimal expression on CD34+CD38− HSCs and lymphoid progenitor cells^107^^,^^108^	expression in myeloid-lineage cells pose a risk for severe myelotoxicity.^107^^,^^108^ CLEC12A^neg^ cells have been observed in some AML patients^345^	impressive efficacy for CLEC12A-CAR T cells in a phase I trial (70% ORR) but all patients developed severe pancytopenia and two died of severe infection due to chronic agranulocytosis^112^
FLT3	AML bulk cells and LSCs^116^ and B-ALL^118^	HSPCs^346^	expressed in most AML patients, on both bulk cells and LSCs^116^ activating mutations in AML are common and a marker of adverse prognosis^347^^,^^348^ low risk for OTOT toxicities: healthy tissue expression restricted to a subset of HSPCs^116^ pro-survival role in HSPCs suggests a reduced risk for antigen escape/loss^346^	expression on HSPCs could lead to profound myelosuppression^346^ cytoplasmic expression has been detected in the cerebellum^346^	FLT3 is overexpressed in KMT2Ar B-ALL. KMT2Ar B-ALL can lineage switch (ALL to AML) as a means of antigen escape to lymphoid-targeting therapies. FLT3 CAR T cells may provide therapeutic benefit in the lineage-switch setting^118^ clinical trials are ongoing with results pending
ILT3 (LILRB4)	monocytic AML^121^^,^^340^	monocytes^121^^,^^340^	expression in highly restricted to the monocyte lineage, with no expression on HSCs or on non-hematopoietic cells^340^ highly and homogenously expressed in monocytic AML, with variable partial expression in myelomonocytic AML^121^^,^^340^ expressed on immunosuppressive cells. Eliminating these cells may enhance anti-tumor efficacy^121^ immunoinhibitory and promotes AML migration and infiltration^121^	restricted to a subset of AML^121^^,^^340^ expressed on monocytic cells may result in monocytopenia^121^^,^^340^	a first-in-class myeloid checkpoint inhibitor ILT3-blocking antibody has been developed and a phase I study is underway (NCT04372433)
IL1RAP	AML bulk cells and LSCs^126^	monocytes and epithelial cells^349^	expressed on both bulk cells and LSCs^126^^,^^350^ pro-survival role in AML^126^^,^^351^ not expressed on HSPCs^126^	approximately one-third of patients do not express IL1RAP^350^^,^^352^ expression on monocytes and epithelial cells poses a risk for monocytopenia and endothelial cell damage (which can aggravate CRS)^349^	also a target of interest for CML^353^
CD70	AML bulk cells and LSCs^125^ DLBCL, FL, HL, WM, and MM ^306^	subset of activated B and T cells and dendritic cells ^306^	pro-leukemic role, which may reduce the risk for antigen escape/loss^354^ not expressed on HSPCs and only transiently expressed on a subset of hematopoietic cells^125^	variable expression in AML^125^	CD70 expression is upregulated by the hypomethylating agent azacitidine (already used clinically in AML), providing a rationale for combination therapy^355^
CD44v6	MM and AML (FLT3/DNMT3A mut)^122^	keratinocytes, skin, and oral mucosa. Circulating monocytes and T cells^122^^,^^190^	not expressed on HSCs^122^ pro-leukemic role, which may reduce the risk for antigen escape/loss^122^	variable expression in AML^122^ expression on monocytes poses a risk for modest monocytopenia^122^ transient expression on activated T cells may result in CAR-T cell fratricide^190^	a phase I/II trial (NCT04097301) for CD44v6 CAR T cells in AML or MM was terminated early due to lower-than-expected proportion of patients expressing CD44v6 (Clinicaltrials.gov, accessed 02.03.2024)
Folate receptor β	AML^123^	myeloid-lineage cells^123^	not detected on adult HSCs^123^	expression on monocytes poses a risk for monocytopenia^123^ variable expression in AML^123^	–
GRP78	AML^124^	none under normal conditions	only expressed at the cell surface during ER stress, so should be absent on healthy cells^124^	variable expression in AML and little to no expression LSCs^124^	also a target of interest for myeloma^202^

a CML, chronic myeloid leukemia; HPCs, hematopoietic progenitor cells; HSCs, hematopoietic stem cells; LSCs, leukemic stem cells. Other abbreviations as in Tables 1 and 2.

Owing to its high and homogeneous expression in AML, including on the cancer-repopulating leukemic stem cells (LSCs),^84^^,^^85^ CD33 has long been established as a therapeutic target for AML. Although concerns over fatal cytopenias and hepatotoxicity, as seen with the CD33-ADCs, prompted caution,^86^^,^^87^^,^^88^ CD33 has been extensively investigated clinically as a TCRT target and initial results suggest that hepatic toxicity may be uncommon and cytopenias manageable.^89^^,^^90^^,^^91^ An initial CD33-CAR-T cell phase I trial illustrated the challenges of lymphopenia, a common occurrence in AML that can impede autologous CAR-T cell manufacturing and efficacy, with just three of the 10 enrolled patients in one study able to receive the CAR-T product.^91^ A more recent phase I/II trial, in the pediatric setting, showed manufacturing feasibility, successfully producing CAR-T therapies for 23 out of 24 enrolled patients.^92^ However, CD33-TCRTs have failed to recapitulate the impressive anti-tumor responses seen with CD19- and BCMA-TCRTs and efficacy has been limited.^89^^,^^91^^,^^92^^,^^93^^,^^94^

With its pro-survival role in AML, CD123 is an attractive target.^95^ Although monocyte and granulocyte expression pose a concern, low expression on hematopoietic progenitors may mitigate the risk of prolonged, severe myelosuppression.^96^^,^^97^^,^^98^ Indeed, this was confirmed in a phase I study, with arguably lower-than-expected levels of severe cytopenia.^99^ Unfortunately, the efficacy seen in this and other clinical studies of CD123 bispecific TCEs has not been impressive.^99^^,^^100^^,^^101^ Preliminary results from clinical trials suggest that CAR T cells may be a more effective modality for targeting CD123,^102^^,^^103^ but potentially at the cost of safety. Two patients in the Cellectis UCART123 trial developed grade 4 capillary leak syndrome, and one experienced grade 5 cytokine release syndrome (CRS).^104^^,^^105^ These serious adverse events may well be due to endothelial CD123-expression, which can be increased by interferon gamma (IFNγ) and tumor necrosis factor alpha (TNFα) during CRS, further exacerbating endothelial damage and CRS in a positive feedback loop.^106^

CLEC12A expression is mostly restricted to the myeloid compartment with minimal expression on CD34+CD38− HSCs and lymphoid progenitor cells,^84^^,^^107^^,^^108^ suggesting that CLEC12A targeting would not completely impair patients’ normal hematopoietic potential. Preclinical studies supported this, with retained progenitor cell function permitting count recovery after transient cytopenias.^109^^,^^110^^,^^111^ However, as is often the case with TCRT studies, the preclinical data were not predictive of clinical outcome. Chronic myelosuppression was a consistent feature in a phase I trial of a CLEC12A CAR T cell, and at least two deaths from infection in the setting of chronic agranulocytosis were reported.^112^ Nevertheless, severe myelosuppression is a general feature of all salvage therapies for AML, and response rates in this and a second phase I trial in pediatric AML were impressive, allowing a bridge to transplant.^113^ Early-phase trials with TCEs suggest more manageable myelosuppression, but perhaps at the expense of clinical response.^114^^,^^115^

Unlike CD33, CD123, and CLEC12A, FLT3 off-tumor expression is largely confined to a subset of HSPCs and is much lower than on malignant cells, which may provide a window for targeting AML without profound myeloablation.^116^ Preclinical studies attempting to gauge the degree of myelosuppression have been mixed, with some suggesting preserved stem cell numbers and function,^117^^,^^118^ and others suggesting that prolonged cytopenias are likely to be a feature.^119^^,^^120^ These differences almost certainly reflect differences in the models used, and it would seem prudent to assume that marked cytopenias are likely. Clinical trials of bispecific TCEs and CAR T cells are underway, but outcome data are not available at the time of writing. FLT3 is also highly expressed in KMT2Ar acute lymphoblastic leukemia (ALL), a disease that has been shown to undergo lymphoid-to-myeloid lineage switch following CD19-CAR-T cell therapy as a mechanism of antigen escape.^118^ Thus, FLT3 TCRTs may be beneficial to both treat and prevent lineage-switch relapses.^118^

Other targets of interest for AML that show minimal or no expression on HSPCs, and therefore may preclude the use of allo-HSCT, include ILT3 (LILRB4), CD44v6, folate receptor β, and GPR78 (Table 3).^121^^,^^122^^,^^123^^,^^124^ However, variable inter- and intra-patient target expression may limit therapeutic utility and increases the risk for antigen-negative/low relapse. Pro-leukemic proteins, such as CD70 and IL1RAP (Table 3), may also prove useful.^125^^,^^126^ However, given the challenges of targeting AML, it is likely that successful TCRTs will require some of the alternative engineering strategies discussed later on in this review.

### T cell malignancies

T cell leukemias and lymphomas encompass a broad spectrum of phenotypically mature and immature neoplasms that can arise at any stage during T cell development. While prognosis can vary greatly between subtypes, even favorable subtypes adopt a very poor outlook in the relapsed-refractory setting.^127^^,^^128^ Contrary to B cell malignancies, the development of immunotherapies for T cell neoplasms has been slow. As with AML, a major challenge is the lack of tumor-unique antigens. B cell aplasia can be clinically managed using immunoglobulin infusions, but no equivalent therapy exists to replace T cell function. A further challenge, unique to T cells, is that target antigens are frequently shared by the effector CAR T cells, leading to CAR-T cell fratricide. This can impact both manufacturing and CAR-T cell persistence *in vivo*.^2^ A third challenge is that, in the autologous setting, the CAR-T infusion product could be contaminated with malignant cells. Nevertheless, these challenges are now being addressed, and there are several ongoing TCRT clinical trials. Most of these are against lineage-specific antigens and thus rely on the use of allo-HSCT or CAR-T cell suicide switches to reverse T cell aplasia. A summary of potential T cell targets is presented in Table 4.

**Table 4:** Preclinical and early-phase TCRT targets for T cell malignancies

Target^a^	Reported on-tumor expression	Reported off-tumor expression	Advantages	Disadvantages	Additional notes
CD7	T-ALL/LBL, subset of PTCL^2^	T, NK cells and B and myeloid-cell progenitors^356^	highly expressed on the majority of T-ALL/LBL, and a subset of peripheral T cell lymphomas^2^ OTOT toxicities limited to the hematopoietic compartment^356^	healthy T cell expression results in severe CAR-T cell fratricide without modification. These modifications can complicate CAR-T cell manufacturing^2^ OTOT expression results in the short-term ablation of T- and NK cells, which may increase the risk of infections^131^ CD7 antigen escape post CD7-targeted TCRT has been reported^131^^,^^132^	clinical trial results suggest that CD7^neg^ healthy T and NK cells can expand to reconstitute the immune system post CAR-T cell infusion^129^^,^^132^^,^^357^ CD7-CAR T cells have shown high CR rates across multiple trials but also high rates of severe infections^129^^,^^130^^,^^131^^,^^132^^,^^133^^,^^308^ also a target of interest for some AML ^358^
CD5	T-ALL, T-lymphoma, and some B cell malignancies ^135^	thymocytes, peripheral T cells, and some B cells^135^	expressed in the majority of T-ALL and T cell lymphomas^135^ CD5-CAR T cells have shown efficacy in CD7^neg^ patients post CD7-CAR-T cell therapy^139^ CD5 negatively regulates T cell activation to prevent overactivation and activation-induced cell death. Thus, KO of CD5 in CAR T cells may minimize fratricide and also enhance anti-tumor efficacy^359^ OTOT toxicities limited to hematopoietic compartment^135^	CD5 is expressed on normal T cells, which poses a risk for CAR-T cell fratricide and increased exhaustion.^359^ Preclinical studies suggest expression is reduced during CAR-T cell manufacturing and that this isn’t a concern^135^^,^^136^ OTOT expression poses a risk T cell aplasia.^139^ Thus far, however, prolonged complete T cell aplasia have not been observed^137^^,^^138^^,^^139^	early results from CD5-CAR-T cell trials suggest efficacy^139^^,^^360^
CD4	mature T cell lymphomas and some T-ALL^140^^,^^142^	most T cells (helper and regulatory T cells)^361^	highly expressed in most mature T cell lymphomas (PTCL and CTCL) and some T-ALL^140^^,^^142^ OTOT toxicities limited to the hematopoietic compartment. CD4 is not expressed on HSCs so CD4+ T cell depletion could be reversed^140^ Targeting of Tregs may enhance anti-tumor efficacy^142^	prolonged CD4+ T cell aplasia can be fatal secondary to opportunistic infections^141^ CD4 expression on normal T cells leads to CD4+ CAR-T cell fratricide.^140^ CD4+ cells may be important for long-term responses^362^ CD4^neg^ relapse was seen in a preclinical model^363^	clinical trials are ongoing with limited results, but early reports suggest efficacy^142^
CD30	HL, variable expression in NHL (both B and T cell), including DLBCL, ALCL and CTCL, and T-ALL ^364^^,^^365^^,^^366^	activated HSPCs, T cells, B cells, and NK cells^364^^,^^365^^,^^367^ skin keratinocytes^368^	simultaneous elimination of CD30^pos^ alloreactive T cells may minimize the risk of graft rejection when using allogenic CD30-CAR T cells^369^ CD30 plays an immunoregulatory role. Loss of CD30 on CAR T cells (such as by fratricide) may improve anti-tumor activity^370^ apart from a subset of activated immune cells, CD30 expression is highly restricted^365^	CD30 is transiently upregulated on activated B, T, NK cells, and HSPCs, which could pose a risk for OTOT toxicities. Preclinical studies suggest that the differential expression between these cells and tumor cells may provide protection.^364^^,^^367^ Cytopenias from CD30-CAR-T cell trials seem to be self-limiting and the risk of infections is low^368^^,^^369^^,^^371^ expression on skin keratinocytes may be the cause for reported transient skin rashes^368^^,^^371^	clinical experience with brentuximab-vedotin (CD30-ADC) in HL suggest that CD30 can be safely targeted.^372^ CD30-CAR T cells show similar safety and promising efficacy, although numbers of patients with T cell malignancies is low^368^^,^^369^^,^^371^^,^^373^^,^^374^ CD30-CAR T cells with transgenic CCR4 expression are currently being trialed in R/R CD30+ HL and CTCL, which may improve migration toward the tumor cells.^374^ Preliminary results support the use of CCR4 to improve tumor localization
TRBC1/2	mature T cell malignancies (PTCL, AITL, T-PLL, ATLL, CTCL) and some T-ALL^143^^,^^375^	approximately one-third to two-thirds (TBRC1/TRBC2) of healthy T cells^143^	αβ TCR expressed in majority of PTCL-NOS and AITLs and approximately 30% of T-ALL^143^ clonal malignant T cells would be depleted, while sparing 35% or 65% (TRBC2 or TRBC1) of the normal T cell repertoire^143^ Contaminating malignant cells can be easily identified and removed^376^	CAR-T cell fratricide during manufacturing could limit persistence post-infusion^376^ targeting the TCR may result in bidirectional killing (target cell mediated killing of CAR T cells and other healthy T cells), limiting CAR-T cell persistence and efficacy^377^	a TRBC1-CAR-T cell trial is currently ongoing (NCT03590574) and preliminary results suggest durable responses^144^
TRBV	mature T cell malignancies (PTCL, AITL, T-PLL, ATLL, CTCL, T-LGLL, and some T-ALL)^143^^,^^147^^,^^375^	small subset(<10%) of healthy T cells^146^	clonal malignant cells would be depleted while sparing most of the T cell repertoire^375^^,^^378^ minimal fratricide (depending on disease burden)^146^ the lower frequency of antigen-positive healthy cells may reduce the amount of bidirectional killing of healthy CAR^pos/neg^ T cells^375^^,^^378^ contaminating malignant cells can be easily identified and removed^146^	heterogeneity could limit the therapeutic applicability, although some variable segments appear to be more frequently used in some subtypes^147^ potential risk for cross-reactivity between similar variable genes^375^	TRBV9 mAbs are being currently trialed in axial spondyloarthritis, which will demonstrate the safety of this approach (NCT05445076 and NCT06333210)
CD1a	cortical T-ALL^149^	cortical thymocytes, Langerhans cells, and a subset of myeloid DCs^149^	not expressed on healthy mature T cells; therefore, little risk for CAR-T cell fratricide and T cell aplasia^149^ contaminating malignant T cells can be easily removed from the apheresis product by selecting for CD1a-negative cells^149^	target expression restricted to a subset of T cell malignancies (30%–40% of T-ALL)^149^ OTOT toxicities: Langerhans cell expression may pose a risk for skin-related AEs and loss of cortical thymocytes may compromise immunity^149^	Two CD1a-CAR-T cell trials are currently underway (NCT05745181 and NCT05679895)
CD37	PTCL^30^	healthy B cells and minimal monocyte expression^30^^,^^288^	Is not expressed on T cells, so no risk for CAR-T cell fratricide or T cell aplasia^30^^,^^288^	off-tumor expression will result in B cell aplasia^288^ variable expression in PTCLs^30^	CD37-CAR-T cell trials are ongoing. One patient with CTCL achieved a deep response^36^
CCR9	T-ALL ^152^	small subset of healthy T cells and B cells (<5%), thymocytes, and gut-resident immune cells^152^	expressed in the majority of T-ALL and only a subset of healthy T cells, minimizing risk of T cell aplasia^152^ contaminating malignant cells could be easily identified and removed	CCR9 is unessential, which may pose a risk for antigen-negative/low clone escape^152^ expression on gut-resident immune cells and thymocytes may compromise immunity^152^	CCR9 small-molecule inhibitors have been trialled in Crohn’s disease, suggesting potential safety for this target^152^
UMG1 (unique epitope of CD43)	cortical T-ALL and variable expression in other T-ALL subsets^153^	small subset of healthy T cells (<5%) and cortical thymocytes^153^	low risk for on-target toxicities and fratricide as minimal off-tumor expression (cortical thymocytes and a small subset of circulating T cells)^153^ low risk for fratricide^153^ contaminating malignant cells can be easily identified and removed	target predominantly expressed by cortical T-ALL, with variable and mostly minimal expression in other subsets^153^	may also be a target of interest for DLBCL^153^

a AITL, angioimmunoblastic T cell lymphoma; ALL/LBL, acute lymphoblastic leukemia/lymphoblastic lymphoma; ALCL, anaplastic large-cell lymphoma; CTCL, cutaneous T cell lymphoma; PTCL, peripheral T cell lymphoma; PTCL-NOS, peripheral T cell lymphoma not otherwise specified; T-LGLL, T cell large granular lymphocytic leukemia; T-PLL, T cell-prolymphocytic leukemia. Other abbreviations as in Tables 1, 2, and 3.

CD7 is one of the most advanced targets for T cell malignancies. Attempts to mediate T cell fratricide include knockout of CD7 in the CAR T cells,^2^^,^^129^^,^^130^ sequestration of CD7 in the cytoplasm,^131^^,^^132^ and ignoring it altogether in a “survival-of-the-fittest” approach.^133^ CD7 allogeneic and autologous CAR T cells employing these approaches have achieved good clinical outcomes.^129^^,^^130^^,^^131^^,^^132^^,^^133^ Some, but not all, relapses were with CD7^neg^ disease,^130^^,^^131^^,^^132^ suggesting that both limited CAR-T cell persistence and antigen escape are responsible for treatment failure. Loss of endogenous T cells was also a common feature of these trials and predictable infections, such as Epstein-Barr virus (EBV) and cytomegalovirus reactivation, were seen.^129^ In some cases, these infections proved fatal.^129^^,^^130^^,^^131^^,^^134^ Although some patients remained in remission and recovered their blood counts,^130^^,^^131^^,^^132^ it seems likely that CAR-T cell targeting of CD7 will be best employed as a bridge to transplant.

CD5 is another pan-T cell antigen, but, unlike CD7, expression is reportedly reduced at the cell surface on CAR T cells during manufacturing, limiting fratricide and permitting full expansion.^135^^,^^136^ Whether this is due to downregulation, masking, or sequestration is unclear, but it does not occur on target cells and there is no evidence of antigen escape preclinically.^135^^,^^136^ Clinical trials are somewhat in their infancy but suggest that CD5-CAR T cells are effective.^137^^,^^138^ Numbers are small, but there is a hint that efficacy and toxicity may be correlated; biepitopic targeting was associated with both deeper responses but also greater immune suppression and one grade 5 EBV infection.^139^ It is too early to comment on long-term toxicity, but again it is likely that CD5 CAR T cells will prove most useful as a bridge to transplant.

CD4 is a well-described TCR co-receptor expressed by helper and regulatory T cells and expressed in most mature T cell lymphomas and some T-ALL subsets.^140^ As CD4 is not expressed on HSCs, depletion may be reversible, reducing the risk for prolonged CD4 T cell aplasia, which can be fatal secondary to opportunistic infections.^141^ Preclinically, CD4+ CAR-T cell fratricide was prominent but preliminary results from phase I dose-escalation study suggest that CD4-CAR T cells are effective and that CD4+ T cell recovery is possible.^142^

While these target antigens have demonstrated promising efficacy, healthy T cell aplasia, even if transient, remains problematic. The TCR may offer a more specific way to target neoplastic T cells. Most T cells express an alpha and a beta TCR chain, with the constant region of the latter encoded by one of two genes: *TRBC1* or *TRBC2*.^143^ As T cell malignancies are clonal, targeting one of these two proteins would deplete all the malignant cells but leave a substantial part of the normal T cell repertoire intact (∼35%–65%).^143^ Early results from a phase I/II dose-escalation study for TRBC1-CAR T cells support this theory, with modest, transient, and tolerable drops in T cell counts post infusion.^144^ Given the larger number of variable gene segments for the TCR beta chain, TRBV-TCRTs would similarly eliminate clonal tumor cells but spare a much larger proportion of the healthy T cells (>90%).^145^ Inter-patient heterogeneity could prove challenging, but some malignancies do demonstrate a degree of recurrent expression of certain segments, such as Vβ2, Vβ5, and Vβ8.^146^^,^^147^ Targeting the variable region for neoplasms is currently at the preclinical stage, but ongoing clinical trials with a TRBV9 monoclonal antibody (mAb) in axial spondyloarthritis will help validate the safety of this approach.^148^

More specific targeting may also be achieved using CD1a, CD37, CD30, CCR9, and UMG1 (Table 4), albeit with more limited therapeutic applicability. Cortical T-ALL, a major T-ALL subtype, comprising 30%–40% of disease, is characterized by CD1a expression.^149^ CD1a has minimal off-tumor expression, with no expression on mature healthy T cells. Consequently, neither CAR-T cell fratricide nor T cell aplasia are concerns, although targeting healthy cortical thymocytes may compromise immunity to some extent.^150^ CD37 is aberrantly expressed in some T cell lymphomas but not in resting or activated healthy T cells,^30^ and CD30 is similarly expressed in some T cell lymphomas with only transient expression in activated healthy T cells.^151^ Clinical trials for CD1a-, CD30-, and CD37-CAR-T cell trials are currently underway. CCR9 and UMG1 are still at the preclinical stage, but have both shown promise for the more specific targeting of T-ALL and cortical T-ALL respectively.^152^^,^^153^

## Novel targeting strategies to improve TCRT tumor specificity

As mentioned above, CD19- and BCMA-targeting TCRTs are tolerated because loss of the healthy counterpart cells can be managed clinically. However, for other hematological malignancies, shared expression of target antigens with healthy cells poses a serious safety concern. Even prolonged B cell aplasia is not without consequence.^39^ In addition, even targets with acceptable off-tumor expression profiles may be less restricted than initially thought, leading to unexpected and severe on-target/off-tumor toxicities.^57^^,^^62^^,^^104^^,^^105^^,^^154^ In the second part of this review, we discuss alternative antigens and engineering strategies that aim to mitigate these toxicities and expand the clinical success of TCRTs.

### Targeting neoantigens

Antibody and CAR-T cell targets are classically proteins expressed in their native conformation on the cell surface of a tumor. A neoantigen, on the other hand, is a peptide derived from a mutant protein (which may represent either a driver or passenger mutation) and presented by major histocompatibility complex (MHC) molecules to promote T cell engagement, expansion, and cytolysis. These tumor-unique mutations provide high specificity and, as they can arise from intra- and extracellular proteins, greatly expand the number of potential targets for TCRT therapy.^155^ The potential for neoantigens as immunotherapy targets arose from the realization that the clinical successes of tumor-infiltrating lymphocyte (TIL) therapy, in which patients’ TILs are isolated and expanded *ex vivo* before reinfusion, was in large part due to the presence of T cells reactive against somatic mutations present in the tumor.^156^^,^^157^ Although encouraging responses have been seen with TIL approaches, relapse, likely due to T cell exhaustion, appears to be the norm.^158^^,^^159^

#### TCR T cells and TCR-mimics

To improve clinical efficacy, neoantigen-reactive TCR sequences can be identified from patients and then cloned into healthy, naive T cells (TCR T cell), similar to CAR-T cell therapy. Neoantigens can also be targeted using antibodies specific for peptide-human leukocyte antigen (HLA) complexes, known as TCR-mimics. These TCR-mimics can be used as the antigen-recognition domain in CAR T cells or in bispecific TCEs,^160^^,^^161^^,^^162^ combining the specificity of TCR T cells with the simplicity of antibody manufacture.

The majority of neoantigens are unique to an individual’s cancer (i.e., private neoantigens). While offering the potential for individualized therapy, it can be prohibitively costly and labor intensive to develop TCRTs against these.^163^ Public neoantigens, which arise from mutational hotspots, are likely to be shared among multiple patients^164^^,^^165^^,^^166^ and present a more economically viable alternative. For example, Kim et al. recently reported the identification of 39 mutant p53-reactive TCRs.^158^ As p53 is such a common cancer mutation and because several of the identified TCRs paired with prevalent HLA molecules, the authors theorized that this library could be used to treat ∼7% of patients with solid cancers.^158^ Like p53, the RAS family of GTPases, especially KRAS, are frequently mutated in cancer.^165^ These mutations have been shown to be immunogenic^167^^,^^168^ and clinical trials for TCR T cells against KRAS neoantigens in solid tumors have now started recruiting (NCT03190941 and NCT03745326). As KRAS and p53 mutations are also found in hematological malignancies, albeit at a lower frequency, these therapies would likely be of benefit in these settings as well.^169^^,^^170^ Disease-specific recurrent mutations and fusions may provide more public neoantigens for hematological malignancies. TCR T cells reactive against FLT3^D835^, a mutation that occurs in approximately 7%–10% of AML patients, have demonstrated potent and highly selective anti-leukemic activity *in vitro* and *in vivo*.^164^ Other examples of frequent immunogenic neoantigens for hematological malignancies include the NPM1 mutations in AML^171^ and the BCR-ABL fusion protein for Ph-positive ALL.^172^

Phosphopeptides, which are immunogenic and immunologically distinct from parental un-phosphorylated peptides,^173^^,^^174^ further expand the repertoire of targetable neoantigens.^175^ Protein phosphorylation is typically dysregulated in cancer, with an increase in the global number of phosphopeptides presented by the MHC on malignant cells.^175^^,^^176^ Some phosphopeptides are both tumor specific and shared among patients, within and across cancer subtypes,^175^^,^^176^ and TCR T cells targeting these have shown promising preclinical results.^173^^,^^175^^,^^176^^,^^177^^,^^178^ Other post-translational modifications, such as methylation, acetylation, and glycosylation, have also been reported to provide a source of tumor-specific peptides.^179^^,^^180^

Although TCR and TCR-mimic T cells/TCEs enable truly specific cancer targeting, they present their own challenges. Firstly, although some neoantigens may be frequent, they are still not as prevalent as lineage-restricted antigens. Secondly, TCR T cell antigen recognition depends on the HLA allele presenting the peptide. This greatly reduces the number of patients that can benefit from any given therapy^155^ and creates HLA-subtype disparities: HLA-A2 is commonly targeted but this subtype is much more frequent in individuals of European descent compared to other ethnic groups, restricting access to novel therapies for these patients.^181^ Thirdly, although shared neoantigens are often essential for tumor survival, these targets are not immune to antigen escape and MHC loss is a frequent mechanism of resistance.^158^^,^^167^ Finally, while the on-target reactivities of TCRTs against conventional target antigens are relatively easy to predict, the off-target toxicities of TCRs and TCR-mimics, caused by cross-reactivities with other peptide-HLA complexes, are much more unpredictable.^182^^,^^183^^,^^184^ The severe consequences of this were demonstrated by the four patient deaths in two melanoma-associated antigen 3 (MAGE-A3) TCR T cell trials caused by the recognition of neurological MAGE-A12 and cardiomyocyte titin expression.^183^^,^^184^ Predicting these potential off-tumor toxicities is thus crucial but highly challenging.^185^

### Isoforms and alternative splicing

Alternative splicing of pre-mRNA provides proteomic diversity from just one gene and is frequently co-opted by cancer cells, conferring drug resistance, increasing proliferation and/or survival, and inhibiting apoptosis.^186^ Alternative splicing in tumor cells is more prevalent than in normal cells.^187^ It can lead to isoform switching^186^—aberrant expression of normally tissue-restricted isoforms—and intron-retention and splice site neojunctions, which can generate cancer-specific neoantigens.^187^^,^^188^

CD44 is frequently upregulated in hematological and epithelial malignancies, promoting tumor survival and metastasis,^189^ but off-tumor expression (including on HSCs) precludes TCRT development.^122^ Alternative splicing of CD44 produces isoforms with much more restricted expression patterns, such as CD44v6, which is highly, but variably, expressed on AML and myeloma cells but not on HSCs.^122^ CD44v6 CAR T cells demonstrated effective anti-leukemic activity *in vitro* and *in vivo*, with only modest monocytopenia and no toxicity toward HSCs.^122^^,^^190^ Disappointingly, when CD44v6-CAR T cells progressed to the clinic, low patient recruitment rates resulted in an early termination of the trial (clinicaltrials.gov/study/NCT04097301, accessed 02.03.2024).

In addition to creating novel expressed proteins, alternative splicing can generate neoantigens that could represent targets of TCR T cells.^187^^,^^191^^,^^192^^,^^193^^,^^194^ For example, the D393-CD20 splice variant is expressed in some B cell lymphomas, but not resting healthy B cells, and has been shown to be immunogenic.^193^^,^^194^ Recently, circRNAs, back-spliced products of pre-mRNA, have been shown to encode proteins that are tumor specific and shared across patients and that generate immunogenic peptides,^195^^,^^196^^,^^197^^,^^198^ providing another potential source of targetable neoantigens.

### Cancer-specific protein conformations

Altered protein conformations can provide a survival advantage for malignant cells, e.g., by maintaining a receptor in a constitutively active state or confining pro-apoptotic proteins to a non-functional state. However, this altered conformation can also expose unique epitopes that are not normally accessible.^199^^,^^200^ One example of this is ITGβ7 in myeloma. While ITGβ7 is not specific to myeloma, its constitutive activation exposes an epitope that can be recognized by a conformation-dependent CAR T cell that has demonstrated potent and selective killing of myeloma cells *in vitro* and *in vivo*.^199^ A second example is loss of function of the receptor, P2X7, which confers an anti-apoptotic phenotype in many cancers. This non-functional P2X7 has unique epitopes that can be targeted by CAR T cells^200^ and represents a potential pan-cancer target. Cancer-specific conformational changes represent promising targets, but unbiased identification is challenging. Recently, Mandal et al.^201^ reported a structural surfaceomic screen that combined cross-linking mass spectrometry with cell-surface proteomics as a method to identify proteins that are in an altered conformational state. They thus identified and validated ITGβ2 as a novel CAR-T cell target in AML.

### Cancer-specific protein localization

Protein localization is dysregulated in cancer, and exposure of normally intracellular proteins to the cell surface represents another avenue for specific targeting. GRP78 is a regulator of the unfolded protein response that is normally retained in the endoplasmic reticulum (ER) by the binding of its C-terminal KDEL sequence to the KDEL-R1 receptor. Increased ER stress in cancer overwhelms this retention mechanism, leading to translocation of GRP78 to the cell surface in AML, where it can be targeted using CAR T cells.^124^ Cell-surface expression of GPR78 has also been reported for myeloma, and a GRP78-mAb was shown to be well tolerated in a phase I trial, although no objective responses were seen.^202^ Alternative splicing may also alter protein localization, as has been reported for some *ESR1* (ERα) isoforms, thus creating further tumor-specific targets.^203^

While tumor-specific antigens may improve selectivity, innovative CAR designs can also be employed to overcome off-tumor toxicities, challenges of tumor heterogeneity, antigen escape, and limited CAR-T cell efficacy and/or persistence.

### Logic gating

Boolean-logic gating describes the requirement for a CAR T cell to recognize and respond to multiple input signals, minimizing off-tumor toxicities by ensuring that CAR T cells are activated only in the presence of specific combinations of antigens found on the tumor.

#### OR gates (A OR B)

**Figure 2:**
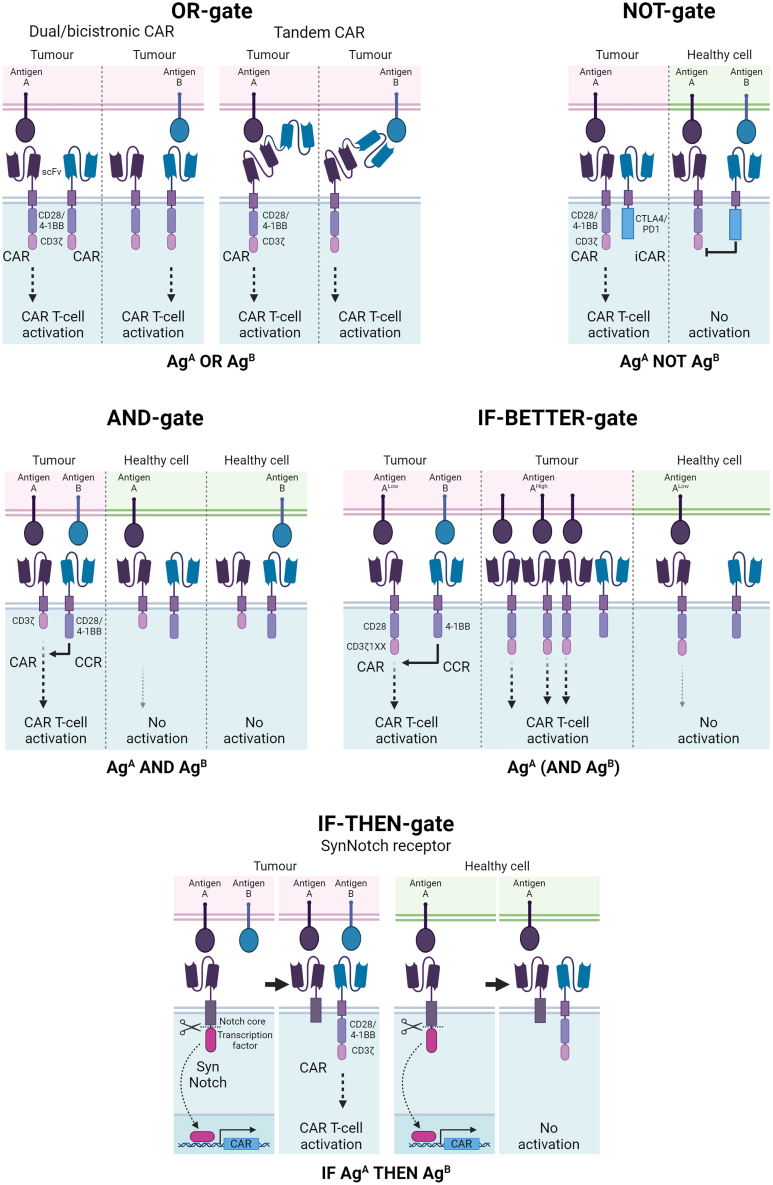
Overview of logic-gated CAR-T cell designs OR gate: CAR T cells may recognize one of two (or more) antigens. Dual or bicistronic CAR designs contain two separate CAR molecules, while tandem CARs contain two scFvs fused to a single stalk. NOT gate: an activating CAR is paired with an inhibitory CAR (iCAR) that contains an inhibitory domain, such as PD-1 or CTLA-4, against a healthy-cell-exclusive target antigen. CAR-T cell activation is inhibited by the iCAR when encountering a healthy cell, while cytolytic activity is maintained against single-target-positive tumor cells. AND gate: a first-generation CAR (does not contain a co-stimulatory domain) is paired with a chimeric co-stimulatory receptor (CCR) that lacks an intracellular signaling domain. Both receptors must be engaged for full target cell activation and effector cell function; therefore, healthy cells expressing only one target antigen are spared. IF-BETTER gate: an attenuated CAR is paired with a CCR. The CCR amplifies CAR signaling to enable T cell effector functions when encountering antigen A^low^ tumor cells. Antigen A^low^ healthy cells that do not express antigen B are spared.^233^ IF-THEN gate: the SynNotch receptor consists of an scFv fused to part of the Notch receptor and a transcription factor. Engagement of the cognate antigen results in the release of the transcription factor, which drives expression of a conventional CAR against a second protein.^234^

Antigen^neg/low^ relapse following CAR-T cell therapy remains a major hurdle to durable remissions. Although tumors often respond to a second targeted therapy, targeting multiple-antigens simultaneous has been shown to be more efficacious and to reduce the risk of relapse.^14^^,^^15^^,^^16^^,^^204^^,^^205^^,^^206^^,^^207^ OR-gated logic enables CAR T cells to respond to one of several antigens (Figure 2). This not only addresses tumor heterogeneity but can enable the simultaneous targeting of immunosuppressive cells within the tumor microenvironment to improve efficacy.^208^^,^^209^ Due to their design simplicity, OR gates are the most clinically advanced logic gates. However, target antigens must still be highly tumor specific, and, as such, OR gates have been predominantly trialed in B cell malignancies^14^^,^^16^^,^^210^ and myeloma.^211^^,^^212^

Despite good response rates, the emergence of single-target-positive cells at relapse suggest that single-antigen targeting may be compromised in OR gates, particularly in tandem CARs. In a CD19/CD22 tandem-CAR trial in large B cell lymphoma and B-ALL (NCT03233854), CD19^neg/low^ relapse was common, consistent with a selection pressure against CD19, whereas CD22 loss or decrease was not seen.^213^ Follow-up *in vitro* studies revealed reduced reactivity against CD22 in the bispecific compared to the monospecific CAR T cells. Other preclinical studies have similarly reported that tandem CARs are superior in eliminating dual-target-positive cells but can have reduced efficacy against single-target-positive cells, likely due to reduced antigen sensitivity.^26^^,^^207^ Thus, optimal OR-gate CAR-T cell effector function requires rational design, with consideration for the length and orientation of each scFv relative to its target antigen.^205^^,^^207^^,^^214^ In some situations, compromised antigen recognition may actually be beneficial: in a BCMA/CD38 tandem-CAR trial for myeloma, the low-affinity anti-CD38 scFv was placed in a sub-optimal orientation to minimize on-target/off-tumor toxicities (ChiCTR1900026286).^212^

Despite initial concerns about increased CRS severity and on-target/off-tumor toxicities from multi-antigen targeting, bispecific CAR T cells for MM and B cell malignancies have been well tolerated with manageable toxicity.^215^

#### NOT gates (A NOT B)

NOT-gated CAR T cells improve tumor specificity by pairing an activating receptor against a tumor-associated antigen with an inhibitory receptor (iCAR) specific to a healthy-cell-exclusive antigen (Figure 2). This system was first demonstrated by Fedorov, who used a PSMA-targeting scFv linked to cytotoxic T lymphocyte-associated protein 4 (CTLA-4) or programmed cell death protein 1 (PD-1) inhibitory domains to selectively inhibit CD19 CAR T cells against PSMA^pos^CD19^pos^ target cells, while maintaining efficacy against PSMA^neg^CD19^pos^ cells.^216^ This reversible inhibition offers advantages over suicide switches by preserving long-term CAR-T cell immunity and is preventive rather than reactive. In addition, NOT-gated CAR T cells may be subject to reduced antigen-dependent exhaustion and CRS.^217^^,^^218^ However, Fedorov also identified a key limitation: the efficiency of the system hinges on the expression level of the inhibitory receptor target and low-density iCAR targets may not sufficiently limit activity.^216^^,^^219^ In some situations, NOT gates can even inadvertently increase cytotoxicity through enhanced avidity and/or target cell engagement,^219^ posing challenges for clinical application. Design adjustments, such as receptor length, stronger or dual inhibitory domains, and increasing the iCAR:CAR ratio, as well as careful target selection to avoid large, bulky, or low-density antigens, could help mitigate these issues.^220^^,^^221^

Probably owing to these challenges, only one NOT-gate CAR T cell has progressed into clinical trials. A2B530, an autologous carcinoembryonic antigen (CEA)-targeting CAR T cell with an HLA-A∗02 inhibitory receptor (Tmod), is in phase I/II trials (NCT05736731) for CEA+ solid tumors with HLA-A∗02 loss of heterozygosity (LOH).^222^^,^^223^ Given that HLA LOH is a common occurrence in solid cancers, this design provides a universal iCAR receptor when targeting multiple tumors.^222^^,^^223^ However, HLA LOH is much rarer in hematological malignancies, which may limit translation in these conditions.^224^ Furthermore, subjecting CAR-T cell therapy to HLA type greatly limits patient eligibility, negating one of the major benefits of CAR over TCR T cell therapy.

In the absence of unique tumor antigens, inhibitory receptors could greatly expand the number of available targets for CAR-T cell therapy and enable the targeting of essential genes to preclude antigen escape.^218^^,^^225^ Although beyond the scope of this review, it should be noted that more progress for NOT-gate CAR NK cells has been made in hematological malignancies and a trial for CD33/FLT3-NOT-EMCN CAR NK cells (SENTI-202) in AML is currently recruiting (NCT06325748).

#### AND gates (A AND B)

AND gates offer another strategy to enhance CAR-T cell precision by requiring dual antigen recognition. A sub-optimal first-generation CAR against one antigen is paired with a chimeric co-stimulatory receptor (CCR), which lacks an intracellular signaling domain, specific to a second antigen (Figure 2). As the simultaneous engagement of both receptors is required to pass the activation threshold for full effector function, healthy cells that share only one antigen with the tumor are protected.^226^

When targeting highly expressed antigens, CCRs can enhance CAR-T cell reactivity toward low-density antigens targeted by the other, first-generation CARs. Hence, AND-gated CAR T cells have been shown to eliminate target^low^ tumor cells that were resistant to standard second-generation CAR-T cell targeting *in vitro*.^227^^,^^228^ Thus, in addition to improving specificity, AND gates can enhance sensitivity. This may reduce the risk for cancer recurrence from antigen^low^ clones and may widen the CAR-T cell therapeutic repertoire by enabling the targeting of low-density antigens that were previously considered unamenable to CAR targeting.

Applying AND gates to more heterogeneous diseases such as AML may be more challenging due to the scarcity of paired tumor-associated antigens homogeneously expressed across the disease.^84^ Nonetheless, provided these antigens are expressed at sufficiently low levels on HSCs, AND gating has the potential to reduce toxicity, even if it is not completely eliminated.^229^ In the absence of shared tumor antigens, Sukumaran et al. proposed targeting tumor-specific patterns. By combining a first-generation anti-PSCA CAR with two hybrid cytokine receptors to convert immunosuppressive transforming growth factor beta (TGFβ) and interleukin 4 (IL4) cytokine signaling into co-stimulatory signaling,^230^ maximal CAR-T cell activity was restricted to the tumor site. This AND-gate design has the added benefit of rendering CAR T cells resistant to these immunosuppressive signals, improving anti-tumor potency and persistence.

Despite their potential, AND gates must be carefully designed to avoid unintended activation and toxicity through the first-generation CAR.^226^ Tousley et al. recently proposed an alternative design to reduce the “leakiness” of the CAR. By replacing the CD3ζ and co-stimulatory domains with the proximal signaling molecules LAT and SLP-76, the authors demonstrated complete abrogation of single-target signaling without compromising efficacy.^231^

#### IF-BETTER

IF-BETTER gates, whereby antigen B enhances the targeting of antigen A but is not obligate for CAR-T cell function, aim to strike a balance between anti-tumor efficacy and selectivity (Figure 2). Using a second-generation CD19 CAR paired with a CD38 CCR with dual-co-stimulatory domains, Katsarou demonstrated the improved targeting of ultra-low-density CD19 tumor cells (∼20 molecules/cell) that were resistant to second-generation CD19-CAR T cells.^232^ While healthy cells expressing CD19 may still be affected, a sufficient difference in antigen density between healthy and tumor cells could offer some protection. This concept was validated by Haubner et al., who employed a similar strategy to target ADGRE2 in AML, an antigen whose expression in HSPCs prevents conventional CAR-T cell targeting. The researchers used an ADGRE2-targeting CAR with limited sensitivity (ADGRE2-CD28ζ1XX) to minimize reactivity against ADGRE2^low^ HSPCs while maintaining strong anti-tumor activity against ADGRE2^high^ AML cells. To counteract the increased risk of escape by antigen^low^ tumor cells—a potential drawback of affinity-tuning strategies—a CLEC12A-CCR was introduced to enhance the elimination of ADGRE2^low^CLEC12A^med/high^ AML cells. CLEC12A^neg^ HSPCs were spared.^233^

#### IF-THEN

IF-THEN gates describe the sequential recognition of two antigens for target cell elimination. The SynNotch receptor design, developed by the Lim lab, is central to most IF-THEN strategies^234^ and is composed of an antigen-recognition domain, the Notch receptor’s core regulatory domain, and a transcription factor (Figure 2). Upon antigen engagement, the receptor is cleaved, releasing the transcription factor to drive expression of a CAR molecule. This theoretically confines CAR toxicity to dual-target-positive cells, and it also enhances efficacy and persistence by limiting antigen stimulation to the tumor site.^235^^,^^236^ However, because of the temporal delay in CAR induction and decay, neighboring single-target cells are not protected.^42^ Thus, antigen selection for IF-THEN pairing requires careful consideration, especially for metastatic or circulating tumors.

This reduced stringency, however, may benefit heterogeneous tumors. Highly specific, but heterogeneous, tumor antigens can “prime” CAR T cells and drive the localized expression of CARs against more homogeneous, but less tumor-specific, antigens. This enables the use of CARs against targets with otherwise unacceptable off-tumor toxicity.^235^ Based on promising preclinical findings, a phase I trial for a epidermal growth factor receptor variant III (EGFRvIII) SynNotch receptor induced EphA2/IL13Rα2 tan-CAR (E-SYNC) in glioblastoma is currently recruiting (NCT06186401) with results pending.

The modular nature of these synthetic receptors means they are highly versatile and can be used to drive multiple outputs, such as the production of immunostimulatory cytokines or therapeutic antibodies to enhance anti-tumor potency, or to initiate apoptotic signals, akin to a NOT gate.^234^^,^^237^ Recently, the Lim lab investigated the potential for the SynNotch system to integrate multiple recognition events to achieve highly specific tumor-targeting using one, two, or even three antigen input AND/OR/NOT gates. The high precision of this approach was demonstrated by the selective killing of CD19^pos^GFP^pos^HER2^neg^ target cells, while sparing CD19^pos^GFP^neg^HER2^pos^ and CD19^pos^GFP^pos^HER2^pos^ cells.^237^ Improved specificity may also be achieved by enhanced antigen-density discrimination. By placing a high-affinity HER2-CAR under the control of a low-affinity HER2-SynNotch receptor, Hernandez-Lopez et al. created “ultra-sensitive” CARs that spared HER2^low^ cells but were more potent against HER2^high^ cells than affinity-modulated CARs.^238^

Although most IF-THEN gates feature the SnyNotch design, alternative systems that use environmental cues to confine CAR expression to the tumor microenvironment exist. This includes the use of a hypoxia-sensing domain to promote CAR degradation under normoxic conditions,^239^ scFvs with pH-restricted target binding,^240^ and masked CARs that are only exposed by proteases exclusive to the tumor microenvironment.^241^

### Pharmacological modulation

For some targets currently undergoing clinical investigation, on-target/off-tumor expression necessitates a suicide switch to rapidly ablate CAR T cells in the event of severe toxicities and/or to restore normal hematopoiesis.^242^ Protein tags, such as CD20 and truncated EGFR, enable antibody-mediated destruction,^242^^,^^243^ although there are potential risks for on-target toxicities from the antibodies themselves.^242^^,^^244^ The inducible caspase 9 (iCasp9) system offers a much faster alternative to the slow kinetics of antibody-mediated killing. The pro-apoptotic caspase9 and drug-binding domain fusion protein triggers rapid apoptosis following dimerization by the small molecule rimiducid. The safety and speed of this approach was first demonstrated in a graft-versus-host-disease setting, in which >90% of the administered modified T cells were eliminated within 30 min, resulting in resolution of symptoms within 24–48 h, and no recurrence.^245^ In CAR-T cell therapy, iCas9 switches have been reported to effectively resolve severe, persistent immune effector cell-associated neurotoxicity syndrome (ICANS).^246^ While effective, the irreversible nature of suicide switches greatly limits CAR-T cell efficacy. Given the high cost, long production time, and limited manufacturing capacity of CAR T cells, this raises significant concerns regarding the long-term practicality of this approach.

**Figure 3:**
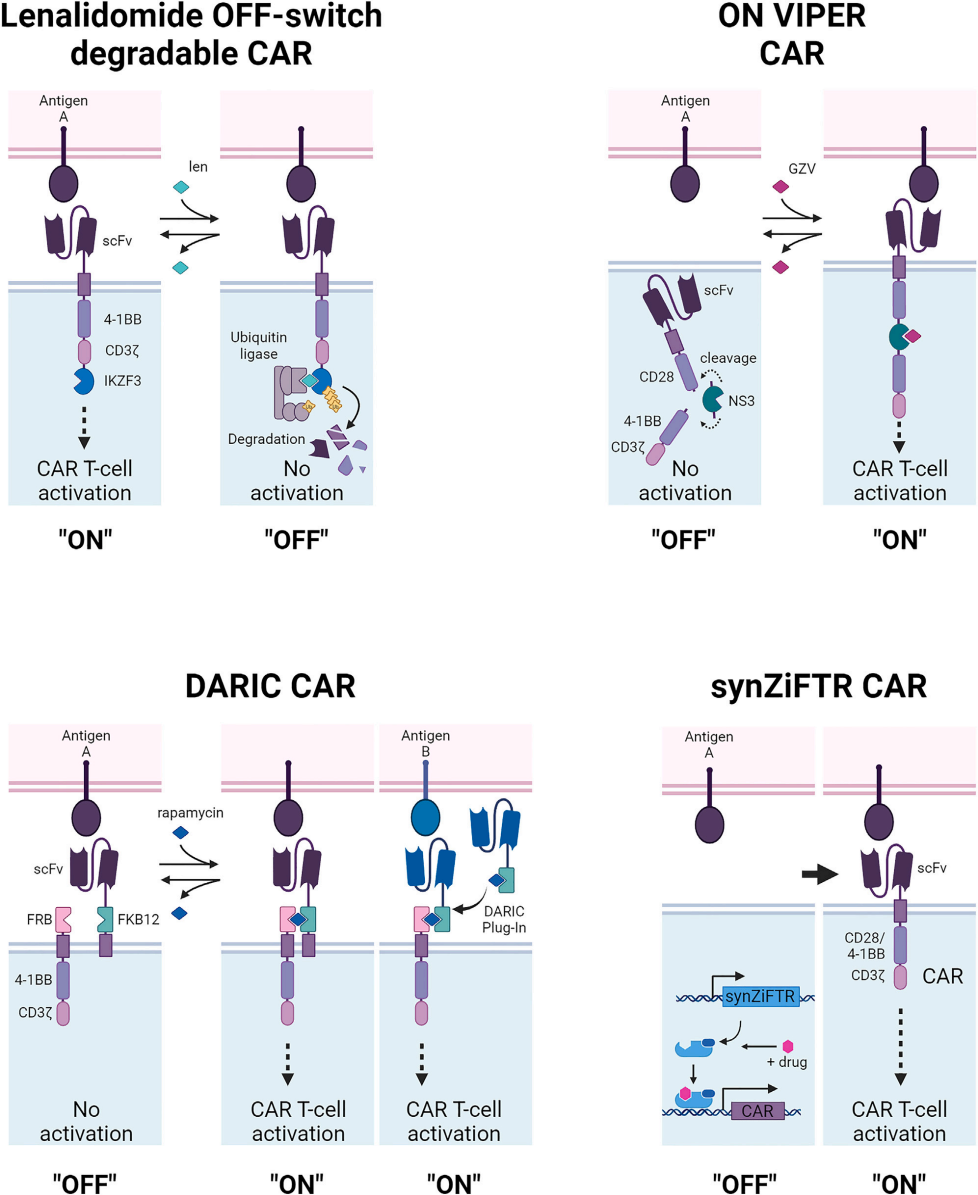
Overview of pharmacologically modulated CAR T cells Lenalidomide-modulated OFF-switch degradable CAR: administration of lenalidomide (len) targets the CAR construct for CRL4^CRBN^-mediated ubiquitination and proteasomal degradation, turning the CAR off.^247^ ON VIPER CAR: versatile protease regulatable CARs contain the hepatitis C virus NS3 protease, which triggers *in cis* proteolysis and CAR fragmentation. NS3 inhibitors (i.e., grazoprevir [GZV]) prevent CAR proteolysis and enable the formation of full-length, signaling-competent CARs.^248^ DARIC CAR: dimerizing agent–regulated immunoreceptor complex CARs are composed of two receptors, one containing the antigen-recognition domain and the other the signaling domain. The small molecule rapamycin induces dimerization of the CAR to form a signaling competent receptor. Rapamycin-prebound scFvs (DARIC Plug-Ins) can redirect the CAR against a second antigen.^250^ synZiFTR: synthetic zinc-finger transcription regulators are DNA-binding zinc fingers fused to drug-binding domains. synZiFTRs, regulated by small molecules, drive the expression of a conventional CAR.^251^

Pharmacological modulation provides a means to reversibly switch CARs on or off, repeatedly, without affecting effector functions. One way to achieve this is to target the CAR for degradation. The incorporation of a zinc-finger degron tag into the CAR construct allows lenalidomide-mediated ubiquitination and proteasomal degradation, effectively creating an “OFF switch” (Figure 3).^247^ Conversely, an “ON switch” can be achieved by inserting hepatitis C virus NS3 proteases into the CAR construct, causing *in cis* proteolysis and CAR fragmentation. Cleavage is inhibited by NS3 protease inhibitors, enabling CAR activation.^248^ Pharmacological modulation can also be achieved by splitting the antigen-recognition and signaling domains and by using small molecules to regulate dimerization.^247^^,^^248^^,^^249^^,^^250^ The extracellular positioning of the drug-binding domain in the dimerizing agent-related immunoreceptor complex (DARIC) ON switch allows the use of exogenous rapamycin-prebound scFvs against additional targets to provide target flexibility.^250^ Synthetic zinc-finger gene regulators fused to drug-binding domains (synZiFTRs) can be used to regulate CAR expression at the transcriptional level. The number of synZiFTRs and small-molecule combinations enables multiplexing, providing temporal control over multiple cellular programs to prime and then activate CAR T cells and optimize clinical efficacy.^251^

An ongoing trial in AML for CD33-CAR T cells employing a DARIC ON switch will help determine the safety and feasibility of incorporating ON/OFF switches clinically (NCT05105152). It is important to note that, although these designs (and the adaptor CARs [AdCARs] described below) can improve CAR safety, they will not improve tumor specificity and cannot completely prevent on-tumor/off-target toxicity.

### AdCARs

**Figure 4:**
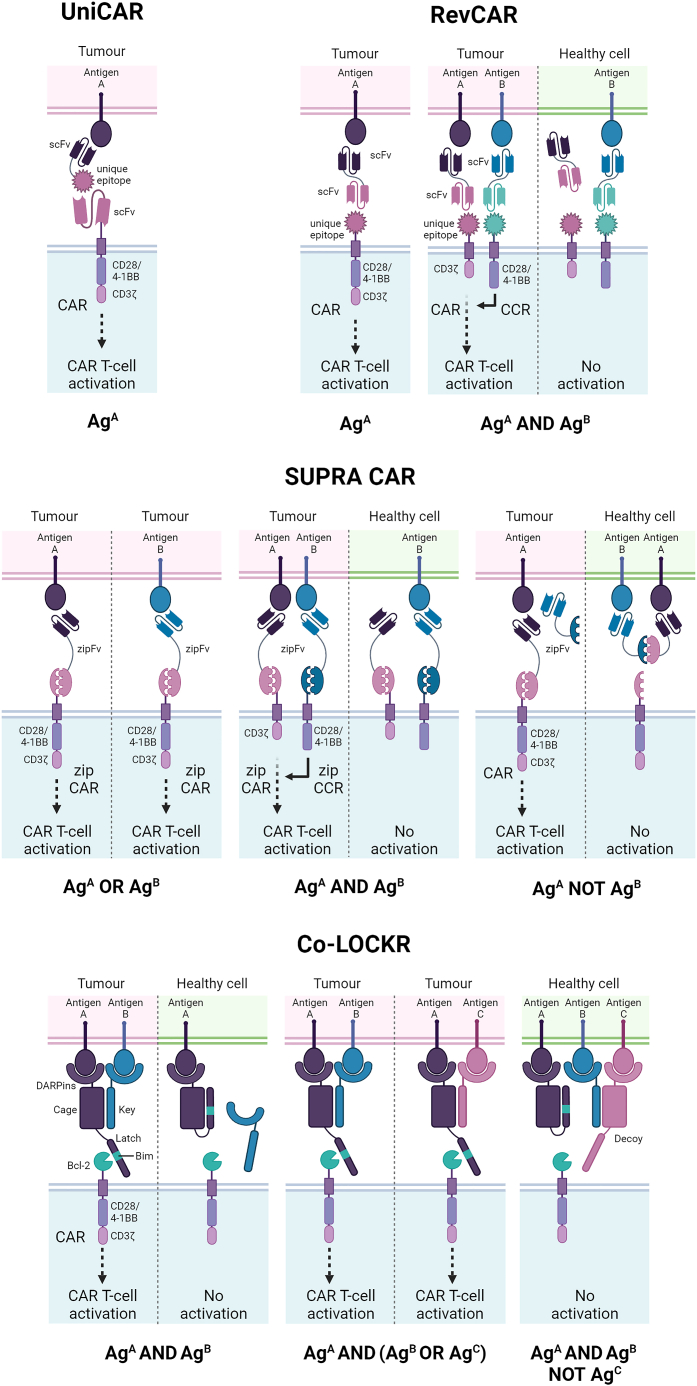
Overview of adaptor CARs The universal CAR (UniCAR) is composed of two components: a universal CAR construct, which recognizes the peptide motif included in the adaptor, and an adaptor containing an scFv against the target antigen. Administration of the adaptor is required for CAR-T cell activation and recognition of the target cell.^256^ RevCAR: bispecific scFvs are used to link the CAR, containing a peptide motif as the extracellular domain, and the target antigen.^262^ The SUPRA CAR design features a zipCAR containing a leucine zipper instead of an antigen-recognition domain, and the cognate leucine zipper fused to an scFv against the target antigen (zipFv). An AND-gate system is created by separating the co-stimulatory and signaling domains and a NOT gate is achieved through competition: when zipFvs against antigen A and B are both engaged, their complementary zippers are engaged, preventing CAR binding.^263^ Co-LOCKR: The colocalization-dependent latching orthogonal cage/key protein system is composed of a “cage” protein that uses a latch domain to sequester a peptide (i.e., Bim) in an inactive conformation. The binding of a key protein triggers a conformational change enabling the peptide to bind to an effector protein (i.e., Bcl-2 fused to a CAR). A second key with a different targeting domain provides an OR gate, while a decoy that sequesters the key can be used to create a NOT gate.^264^

AdCARs utilize adaptor molecules to bridge target antigens and CAR T cells (Figure 4). Similar to pharmacologically modulated CAR T cells, they enable considerable precision over the activation, expansion, and persistence of CAR T cells to reduce on-tumor/off-target toxicity, reduce CRS/ICANS severity, optimize therapeutic efficacy, and limit exhaustion.^252^^,^^253^^,^^254^^,^^255^^,^^256^ They also provide greater flexibility for changing targets without additional manufacturing. AdCARs are particularly promising for hematological malignancies to avoid prolonged hematotoxicity. Provided HSCs are not eliminated by the CAR T cells, normal hematopoiesis can be restored following cessation of adaptor treatment.^257^ This enables the use of CAR T cells against targets such as CD33 and CLEC12A, which would otherwise be restricted to the bridge-to-transplant setting.^254^

Phase I trials of UniCAR T cells with a CD123 adaptor in AML (UniCAR-T-CD123, NCT04230265) provide validation for this hypothesis. Neutrophil counts rapidly recovered following withdrawal of the adaptor with no lasting treatment-induced myelosuppression, and none of the 19 patients required a stem cell transplant for white blood cell reconstitution.^256^^,^^258^ Three grade 3 CRS events were reported, but were all resolved within 24 h of adaptor withdrawal. Importantly, UniCAR T cells could be re-expanded with adaptor re-administration.^256^^,^^258^ A trial for a second AdCAR with a CD123 adaptor is currently recruiting (Allo-RevCAR01-T-CD123, NCT05949125), and a CD19-adaptor CAR for B cell malignancies is also under investigation (CLBR001+SWI019, NCT04450069).^255^

Boolean logic has been applied to AdCARs to further enhance tumor specificity. Split-CAR approaches using dual adaptors against distinct antigens have yielded AND-gated AdCARs for glioblastoma (EGFR/GD2), colorectal cancer (CEA/EpCAM), and AML (CD33/CD123).^259^^,^^260^^,^^261^^,^^262^ The split, universal, and programmable (SUPRA) design provides a NOT-gated AdCAR,^263^ and the colocalization-dependent latching orthogonal cage/key proteins (Co-LOCKR) design impressively recognizes three different inputs using AND, NOT, and OR logic.^264^ These preclinical studies highlight the versatility of logic-gated AdCARs. However, ensuring sufficient bioavailability of the adaptor molecules to balance activating and/or inhibitory signals, already a major hurdle for conventional logic-gated CARs, could preclude clinical translation.

### Generation of tumor-specific antigens

An innovative solution to the lack of truly unique tumor antigens is to create them. Gene editing has already been demonstrated as an effective tool to circumvent CAR-T cell fratricide in T cell malignancies,^265^ but it can also be applied to remove the target antigen or epitope from healthy HSCs *ex vivo*. This renders them immune to CAR- or antibody-mediated destruction and enables the specific targeting of residual cells post HSCT. This approach has been explored in AML to enable the targeting of CD33, an attractive target otherwise limited by the risk of severe myelotoxicity. As demonstrated by Kim and others, CD33 is non-essential, and gene-edited CD33^null^ HSCs are capable of restoring hematopoiesis, while resisting CD33-targeted therapies post transplantation.^266^^,^^267^^,^^268^ Tremtelectogene empogeditemcel (trem-cel, VOR33), a CD33^null^ HSPC product, is currently being trialed in AML in combination with post-transplant gemtuzumab ozogamicin (CD33-ADC) administration, and the results are eagerly anticipated (NCT04849910).

Preclinical trem-cel studies confirmed no significant off-target effects from CD33-gRNA, and many other trials have demonstrated the safety of gene editing.^269^ However, Cas9-induced double-strand breaks can cause chromosomal rearrangements and deletions, risking genetic instability.^270^^,^^271^ Base editing offers a safer alternative and allows for precise epitope engineering of proteins on HSCs to escape CAR-T cell targeting without compromising normal protein function, a requisite for essential genes.^272^ Multiplex epitope engineering enables heterogeneous antigen targeting, further minimizing the risk for antigen^neg/low^ relapse,^273^ and CD123, KIT, and FLT3 have all been proved amenable to this approach.^273^^,^^274^ Epitope engineering also enables the targeting of pan-hematologic antigens, such as CD45, offering a universal therapeutic strategy for hematological malignancies. As CD45 is essential for T cell function, base editing provides a means to disrupt the CD45 epitope without impairing CAR-T cell and HSC function.^275^

Alternatively, introducing unique antigens onto tumor cells is possible with oncolytic viruses. These viruses can specifically infect tumor cells and induce surface expression of CAR-T cell targets with acceptable off-tumor expression profiles, such as CD19 or even GFP.^276^^,^^277^ Oncolytic viruses have innate anti-tumor activity and the release of antigens from lysed cells can contribute to the priming of endogenous T cells against additional tumor antigens in a process known as epitope spreading.^278^ While this tumor-decorating approach is currently at the preclinical stage, oncolytic viruses are under clinical investigation for their role in boosting other TCRT approaches such as CAR T cells and TILs (NCT03740256 and NCT05057715).

## Conclusions

It is likely that, at least with proteins in their natural conformation and location, there is no such thing as a genuinely specific immunotherapy target. Indeed, we have recently reported that this is the case for myeloma.^225^ It is thus probably not a coincidence that TCRTs, and CAR T cells in particular, are most advanced in B cell malignancies and myeloma, where targeting of healthy cellular counterparts is perhaps best tolerated. Targeting of AML and T cell malignancies, where potential on-target, off-tumor toxicity is more pronounced, lags somewhat behind. However, innovative CAR designs are showing considerable promise to achieve more selective targeting in the absence of targets with acceptable safety profiles and it is hoped they will advance TCRT development for these diseases. It is remarkable that many of the successful TCRT targets, such as CD19 and BCMA, were identified on the basis of RNA expression profiles, even though we know that RNA and protein expression are virtually un-correlated at the cell surface.^79^ Cell-surface proteomics is now achieving unprecedented depth and specificity,^79^^,^^80^^,^^82^^,^^84^ but its use for target identification is limited to cancers where relatively homogeneous cell populations can be isolated. Another major challenge, by no means unique to TCRT but particularly pertinent to the field, is the disconnect between preclinical models, especially of toxicity, and clinical outcomes, a point we have argued elsewhere.^279^ Furthermore, the time and expense of conducting clinical trials, means that we need to find more efficient approaches for prioritizing and advancing immunotherapeutic targets. Nevertheless, we believe that this review highlights the considerable drive and ingenuity in this field, and that there are good grounds for optimism in the use of the immune system to target cancer in the future.
